# Palladacyclic Conjugate Group Promotes Hybridization of Short Oligonucleotides

**DOI:** 10.3390/ijms19061588

**Published:** 2018-05-28

**Authors:** Madhuri Hande, Sajal Maity, Tuomas Lönnberg

**Affiliations:** Department of Chemistry, University of Turku, Vatselankatu 2, 20014 Turku, Finland; nimamadhuri@gmail.com (M.H.); sajal.k.maity@utu.fi (S.M.)

**Keywords:** DNA, oligonucleotide, hybridization, organometallic, palladacycle, palladium

## Abstract

Short oligonucleotides with cyclopalladated benzylamine moieties at their 5′-termini have been prepared to test the possibility of conferring palladacyclic anticancer agents sequence-selectivity by conjugation with a guiding oligonucleotide. Hybridization of these oligonucleotides with natural counterparts was studied by UV and CD (circular dichroism) melting experiments in the absence and presence of a competing ligand (2-mercaptoethanol). Cyclopalladated benzylamine proved to be strongly stabilizing relative to unmetalated benzylamine and modestly stabilizing relative to an extra A•T base pair. The stabilization was largely abolished in the presence of 2-mercaptoethanol, suggesting direct coordination of Pd(II) to a nucleobase of the complementary strand. In all cases, fidelity of Watson-Crick base pairing between the two strands was retained. Hybridization of the cyclopalladated oligonucleotides was characterized by relatively large negative enthalpy and entropy, consistent with stabilizing Pd(II) coordination partially offset by the entropic penalty of imposing conformational constraints on the flexible diethylene glycol linker between the oligonucleotide and the palladacyclic moiety.

## 1. Introduction

The groundbreaking discovery of the antitumor activity of cisplatin [1,2] has been followed by efforts to develop more potent anticancer agents based on transition metal complexes [3,4,5,6,7,8,9,10,11]. In particular, problems associated with the presently available platinum anticancer compounds, notably acquired or intrinsic resistance, limited spectrum of activity and relatively high degree of toxicity [12,13,14], have prompted interest in transition metals other than platinum for chemotherapeutic use [4,6,7,8,15]. Palladium is an attractive candidate because its coordination chemistry is similar to that of platinum [16,17]. Pd(II) complexes are, however, kinetically approximately five orders of magnitude more labile than the respective Pt(II) complexes [18]. While the relatively rapid ligand-exchange of Pd(II) should allow formation of thermodynamic (rather than kinetic) products and thus higher selectivity than attainable with Pt(II)-based drugs, it is also likely to result in a different mode of action, at least with simple analogues [19]. No clinically approved palladium-containing drugs are presently available.

The possibility of using palladacyclic complexes as anticancer agents to circumvent the problems caused by the kinetic lability of Pd(II) has received attention over the past decade. The high stability of palladacyclic compounds in physiological media and the resultant low toxicity to normal cells make them promising candidates for future therapeutic agents [9,20]. The selectivity of these agents could be further improved by conjugation to a guiding oligonucleotide. The feasibility of this approach has already been demonstrated with a number of Pt(II)-carrying DNA and PNA oligonucleotides [21,22,23,24,25,26] but with palladacyclic modifications we are only aware of a single recent example, a short DNA oligonucleotide incorporating a single cyclopalladated phenylpyridine residue in the middle of the sequence [27]. In that case, coordination of Pd(II) to the opposite base on a complementary strand was inferred from the abnormally high UV and CD (circular dichroism) signals but the expected stabilization of the double helix by such coordination could not be demonstrated unambiguously. Possibly a stable Pd(II)-mediated base pair was formed but could not be readily accommodated within the base stack, leading to disruption of the double helix.

Herein we describe the synthesis and hybridization properties of short oligonucleotides incorporating cyclopalladated benzylamine “warheads” at their 5′-termini. At monomer level, palladacyclic benzylamine derivatives have already been found to exhibit antitumor activity [28,29]. The 5′-terminal position was chosen to avoid disruption of the double helix by suboptimal coordination geometry. For the same reason, a relatively long and flexible diethylene glycol spacer was used between the cyclopalladated benzylamine and the oligonucleotide.

## 2. Results

### 2.1. Synthesis of the Benzylamine Phosphoramidite Building Block

**Scheme 1:**
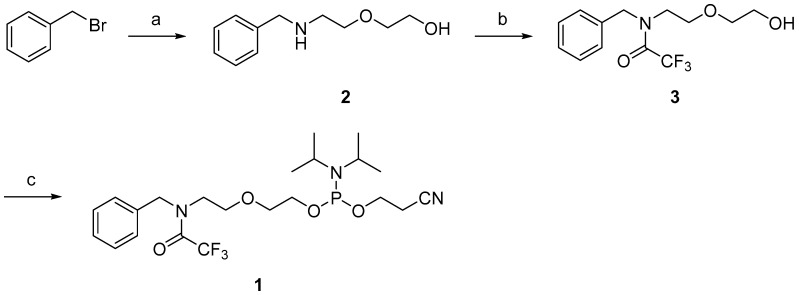
Synthesis of the benzylamine phosphoramidite **1**. Reagents and conditions: (**a**) 2-(2-aminoethoxy)ethanol, MeCN, 25 °C, 16 h; (**b**) ethyl trifluoroacetate, Et_3_N, MeOH, 25 °C, 16 h; (**c**) 2-cyanoethyl-*N*,*N*-diisopropylchlorophosphoramidite, Et_3_N, CH_2_Cl_2_, N_2_ atmosphere, 25 °C, 3 h.

Synthesis of the protected phosphoramidite building block of benzylamine (**1**) is outlined in Scheme 1. First, benzyl bromide was allowed to react with an excess of 2-(2-aminoethoxy)ethanol to give 2-[2-(benzylamino)ethoxy]ethanol (**2**). The secondary amino function was then protected as a trifluoroacetamide by treatment with ethyl trifluoroacetate. Finally, the protected intermediate **3** was phosphitylated by conventional methods to afford the phosphoramidite building block **1**.

### 2.2. Cyclopalladation of 2-[2-(benzylamino)ethoxy]ethanol

**Scheme 2:**
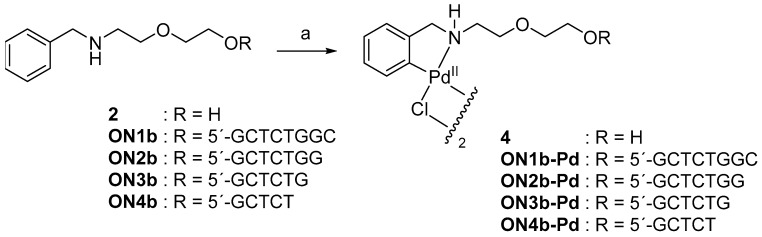
Cyclopalladation of 2-[2-(benzylamino)ethoxy]ethanol (**2**) and the corresponding modified oligonucleotides **ON1b**, **ON2b**, **ON3b** and **ON4b**. Reagents and conditions: a) Li_2_PdCl_4_, MeCN, H_2_O, 25 °C, 16 h.

Cyclopalladation was first tested at monomer level with 2-[2-(benzylamino)ethoxy]ethanol (**2**) by treatment with an equimolar amount of lithium tetrachloropalladate in a mixture of water and acetonitrile. Near-quantitative conversion of the starting material was achieved overnight. ^1^H NMR (nuclear magnetic resonance) spectrum of the product revealed loss of one ortho proton of the phenyl ring and ^13^C NMR spectrum a downfield shift of the respective carbon signal. Both results are consistent with replacement of the ortho proton with Pd(II). The most likely structure of the product is the chlorido-bridged dimer **4** (Scheme 2), as reported previously on related compounds [30,31,32]. While only mononuclear Pd(II) species could be unambiguously identified in the mass spectrum, the splitting of several peaks in both ^1^H and ^13^C NMR is consistent with formation of a dimer, present in both cisoid and transoid forms.

### 2.3. Oligonucleotide Synthesis

The sequences of the oligonucleotides used in the present study are summarised in Table 1. Synthesis of the modified oligonucleotides **ON1b**, **ON2b**, **ON3b** and **ON4b**, having a 5′-terminal benzylamine moiety, was carried out on an automated DNA synthesizer using conventional phosphoramidite strategy. Treatment with concentrated aq. ammonia was employed for removal of the base and phosphate protections and release of the oligonucleotides from the solid support. Cyclopalladation of oligonucleotides **ON1b**, **ON2b**, **ON3b** and **ON4b** was carried out as described above for the monomer **2** (Scheme 2), except that 2.0 equivalents of lithium tetrachloropalladate was used. All modified oligonucleotides were purified by reversed-phase high performance liquid chromatography (RP-HPLC), characterized by electrospray ionization mass spectrometry (ESI-MS) and quantified by UV spectrophotometry.

### 2.4. Hybridization Studies

**Figure 1:**
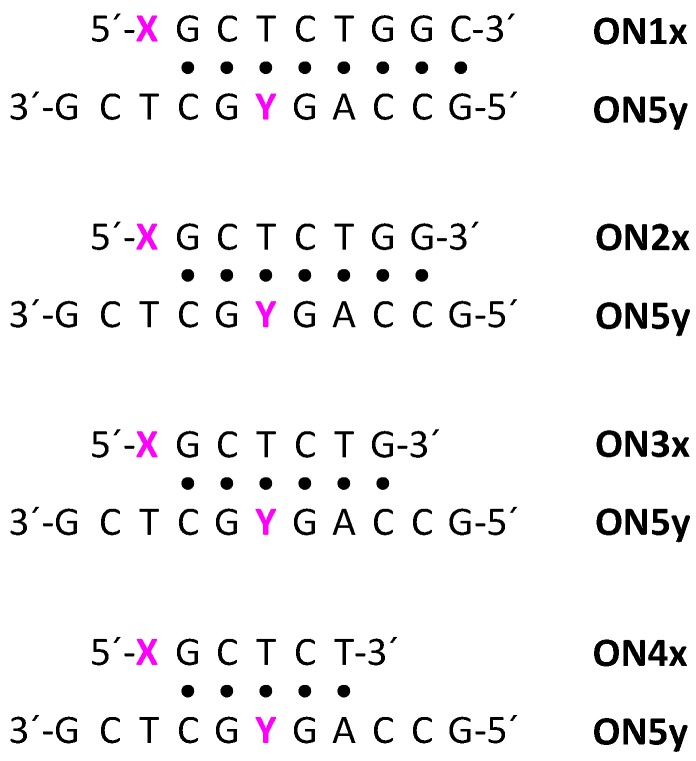
Outline of the hybridization assays used. X is either adenine or unmetalated or cyclopalladated benzylamine and Y is any canonical nucleobase. The bullets indicate Watson-Crick base pairing.

The impact of the 5′-terminal palladacyclic “warheads” on the hybridization properties of short oligonucleotides was assessed by recording melting temperatures of duplexes formed by oligonucleotides **ON1b-Pd**, **ON2b-Pd**, **ON3b-Pd** and **ON4b-Pd** with the natural counterparts **ON2a**, **ON2c**, **ON2g** and **ON2t**. For reference, similar experiments were also carried out on respective duplexes formed by oligonucleotides **ON1b**, **ON2b**, **ON3b**, **ON4b**, **ON1a**, **ON2a**, **ON3a** and **ON4a**, having either an unmetalated benzylamine or an adenine residue at their 5′-termini. In all assemblies, the 5′-terminal residue was placed opposite to a thymine residue of a trinucleotide overhang of the complementary oligonucleotide (Figure 1). A single base pair within the double helical region, on the other hand, was varied to test the sensitivity of hybridization to a single-nucleotide mismatch. All experiments were performed at pH 7.4 (20 mM cacodylate buffer) and ionic strength of 0.10 M (adjusted with sodium perchlorate) and each sample was first annealed by heating to 90 °C and then slowly cooling down to room temperature.

**Figure 2:**
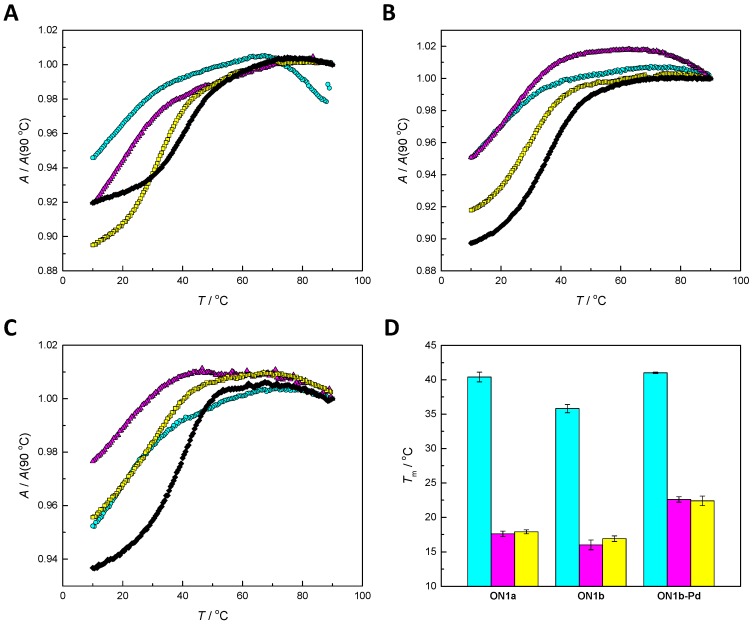
UV melting profiles for duplexes formed by **ON5a** with (**A**) **ON1a** (cyan circles), **ON2a** (magenta triangles), **ON3a** (yellow squares) and **ON4a** (black diamonds); (**B**) **ON1b** (cyan circles), **ON2b** (magenta triangles), **ON3b** (yellow squares) and **ON4b** (black diamonds) and (**C**) **ON1b-Pd** (cyan circles), **ON2b-Pd** (magenta triangles), **ON3b-Pd** (yellow squares) and **ON4b-Pd** (black diamonds); (**D**) melting temperatures of duplexes formed by **ON1a**, **ON1b** and **ON1b-Pd** with **ON5a** (cyan), **ON5g** (magenta) and **ON5t** (yellow); pH = 7.4 (20 mM cacodylate buffer); [oligonucleotides] = 3.0 µM; *I*(NaClO_4_) = 0.10 M. The error bars represent standard deviations of three independent measurements.

The longest matched duplexes **ON1x**•**ON5a** all exhibited sigmoidal melting profiles, with *T*_m_ (melting temperature) values ranging from 36 to 41 °C (Figure 2A–C). The shorter duplexes did not fully hybridize even at the lowest temperature applicable (10 °C) but their *T*_m_ values could still be determined with reasonable accuracy as inflection points of the melting curves. The melting temperatures of the mismatched duplexes, on the other hand, were high enough to be determined reliably only in the case of the longest duplexes **ON1x**•**ON5y**. The A•C mismatch was particularly destabilizing and precluded determination of the T_m_ in all cases, regardless of the length of the duplex. Melting temperatures are summarized in Figure 2D for the longest duplexes and in the Supplementary Materials for all duplexes.

Melting temperatures of the longest matched duplexes **ON1a**•**ON5a**, **ON1b**•**ON5a** and **ON1b-Pd**•**ON5a**, were 40.4 ± 0.7 °C, 35.8 ± 0.6 °C and 41.0 ± 0.6 °C, respectively. In other words, the cyclopalladated benzylamine residue was modestly stabilizing relative to an adenine residue and strongly stabilizing relative to the unmetalated benzylamine residue. To explore the origin of this stabilization, the UV melting experiments were repeated in the presence of 2-mercaptoethanol (100 µM). 2-Mercaptoethanol is a strong ligand for soft transition metal ions and would, hence, be expected to disrupt coordination of Pd(II) to nucleobases. If such coordination is important for duplex stability, a decrease in T_m_ on addition of 2-mercaptoethanol should be observed.

**Figure 3:**
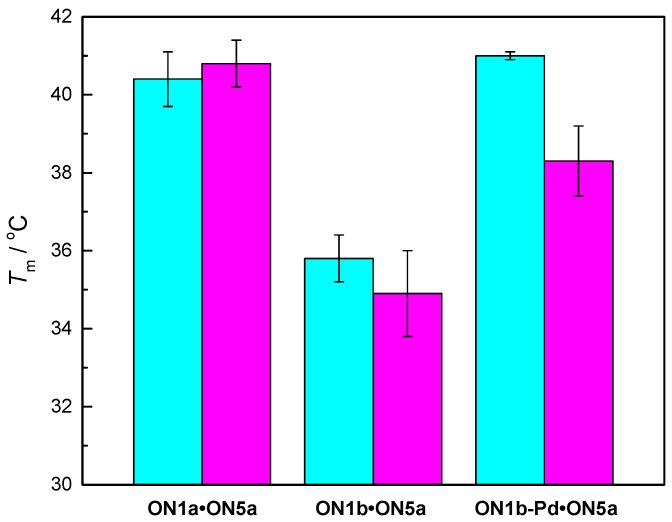
Melting temperatures of duplexes **ON1a**•**ON5a**, **ON1b**•**ON5a** and **ON1b-Pd**•**ON5a** in the absence (cyan) and presence (magenta) of 2-mercaptoethanol; pH = 7.4 (20 mM cacodylate buffer); [oligonucleotides] = 3.0 µM; [2-mercaptoethanol] = 0/100 µM; *I*(NaClO_4_) = 0.10 M. The error bars represent standard deviations of three independent measurements.

Melting temperatures of the longest matching duplexes **ON1x**•**ON5a** in the absence and presence of 2-mercaptoethanol are presented in Figure 3 (all melting temperatures are presented in the Supporting Information). As expected, melting temperatures of duplexes **ON1a**•**ON5a** and **ON1b**•**ON5a** did not change appreciably on addition of 2-mercaptoethanol. With **ON1b-Pd**•**ON5a**, on the other hand, a clear drop in *T*_m_ was observed, consistent with stabilizing coordination of Pd(II) in the absence of competing ligands.

To further elucidate the role of the palladacyclic “warhead”, a detailed thermodynamic analysis of the hybridization of **ON1a**•**ON5a**, **ON1b**•**ON5a** and **ON1b-Pd**•**ON5a** was carried out as described in the literature [33]. The resultant enthalpies and entropies of hybridization are presented in Table 2. With **ON1b-Pd**•**ON5a**, both values were significantly more negative than with **ON1a**•**ON5a** or **ON1b**•**ON5a**.

**Figure 4:**
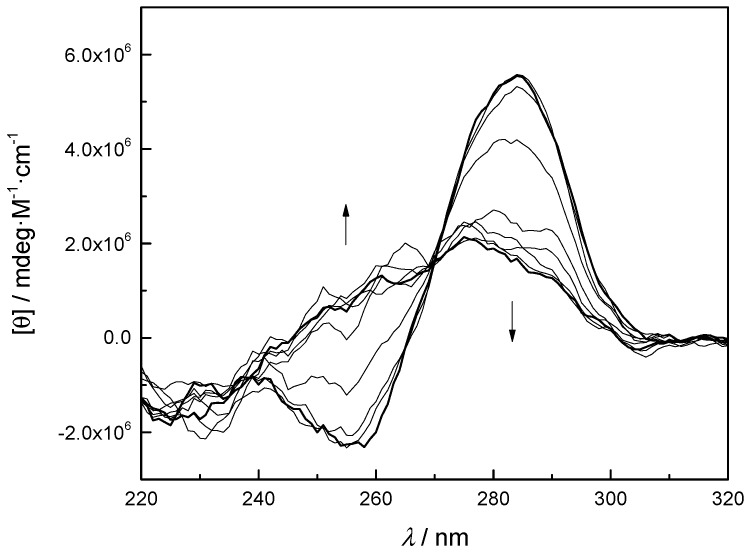
CD (circular dichroism) spectra of **ON1b-Pd**•**ON5a**, recorded at 10 °C intervals between 10 and 90 °C; pH = 7.4 (20 mM cacodylate buffer); [oligonucleotides] = 3.0 µM; *I*(NaClO_4_) = 0.10 M. Spectra acquired at extreme temperatures are indicated by thicker lines and thermal shifts of the minima and maxima by arrows.

Secondary structure of duplexes formed by the cyclopalladated oligonucleotides was studied CD spectropolarimetrically over a temperature range of 10–90 °C at 10 °C intervals. All the other conditions were identical to those of the UV melting experiments. With the longest matched duplexes, the spectra obtained at 10 °C were clearly characteristic of a B-type double helix, with prominent negative and positive signals at 260 and 280 nm, respectively (Figure 4 for **ON1b-Pd**•**ON5a**, all spectra are presented in the Supplementary Materials). Similar, but weaker, signals were observed with the shorter and/or mismatched duplexes, reflecting their lower melting temperatures. In all cases, the signals diminished on increasing temperature, consistent with unwinding of the double helix.

## 3. Discussion

### 3.1. Duplex Stabilization by the Palladacyclic “Warhead”

The sensitivity of the duplex stabilization by the cyclopalladated benzylamine moiety to the presence of 2-mercaptoethanol suggests direct coordination of Pd(II) to a base moiety of the complementary strand. The thymine base directly opposite to the palladacyclic residue appears as the most likely candidate but, given the length and flexibility of the diethylene glycol linker, coordination to other bases of the 3′-overhang cannot be ruled out. The most likely donor atoms within these bases are the N3 of cytosine and thymine and the N1 of guanine [34,35,36].

Duplex stabilization by the cyclopalladated benzylamine moiety is rather modest when compared to stabilizations achieved previously with metal mediated base pairing [37,38,39,40,41,42,43,44,45,46]. One should, however, bear in mind that within a double helix the most stable metal mediated base pairs are formed between two nucleosides or nucleoside analogues, preorganized to place the donor atoms at appropriate positions. In the present case the flexible diethylene glycol linker first has to adopt a conformation conducive to Pd(II) coordination, consistent with the observed highly negative entropy of hybridization. As a result, the stabilizing enthalpic contribution by Pd(II) coordination is almost entirely offset by the entropic penalty.

### 3.2. Impact of the Palladacyclic “Warhead” on Sequence Selectivity

Highly stabilizing modifications are known to be able to override sequence information and allow hybridization of even highly mismatched oligonucleotides [47]. Even in the present case, the mismatched duplexes were actually stabilized more than the matched ones by the cyclopalladated benzylamine moiety. However, the difference in *T*_m_ between the matched and the most stable mismatched duplex (**ON1b-Pd**•**ON5a** and **ON1b-Pd**•**ON5g**, respectively) was still nearly 18 °C, translating into a 10^4^-fold preference of **ON5a** over **ON5g** in hybridization with **ON1b-Pd**.

## 4. Materials and Methods

### 4.1. General Methods

NMR spectra were recorded on Bruker 500 NMR spectrometers (Bruker, Billerica, MA, USA) and chemical shifts (δ, ppm) are quoted relative to the residual solvent peak as an internal standard. Mass spectra were recorded on a Bruker Daltonics microTOF-Q mass spectrometer (Bruker, Billerica, MA, USA). The solvents for organic synthesis were of reagent grade and dried over 4 Å molecular sieves. For preparation of HPLC elution buffers, freshly distilled triethylamine was used. The other chemicals, including unmodified oligonucleotides, were commercial products that were used as received.

### 4.2. N-benzyl-2,2,2-trifluoro-N-[2-(2-hydroxyethoxy)ethyl]acetamide *(**3**)*

2-[2-(benzylamino)ethoxy]ethanol (**2**, 2.00 g, 10.2 mmol) was dissolved in MeOH (10 mL). Et_3_N (2.90 mL, 20.4 mmol) was added and the resulting mixture stirred for 30 min at 25 °C. Ethyl trifluoroacetate (1.46 g, 10.2 mmol) was then added and the reaction mixture stirred for 16 h at 25 °C, after which it was evaporated to dryness to afford the desired product **3** as a mixture of two slowly interconverting rotamers (2.98 g, near quantitative yield). ^1^H NMR (CDCl_3_, 500 MHz, major rotamer): δ 7.19-7.48 (m, 5H), 4.80 (s, 2H), 3.40-3.70 (m, 8H), 2.82 (br, 1H). ^1^H NMR (CDCl_3_, 500 MHz, minor rotamer): δ 7.19-7.48 (m, 5H), 4.82 (s, 2H), 3.40-3.70 (m, 8H), 2.82 (br, 1H). ^13^C NMR (CDCl_3_, 125 MHz, major rotamer): δ 157.1 (q, *J* = 35.8 Hz), 136.3, 128.7, 127.7, 127.6, 116.8 (q, *J* = 285.9 Hz), 72.5, 67.6, 60.9, 50.3, 46.8 (q, *J* = 2.9 Hz). (CDCl_3_, 125 MHz, minor rotamer): δ 156.9 (q, *J* = 35.8 Hz), 135.8, 128.8, 127.9, 127.2, 116.8 (q, *J* = 285.9 Hz), 72.4, 68.9, 61.0, 51.4 (q, *J* = 3.0 Hz), 46.0. HRMS (ESI^+^) *m*/*z* calcd 314.0974 obsd 314.0972 [M + Na]^+^.

### 4.3. 2-[2-(N-benzyl-2,2,2-trifluoroacetamido)ethoxy]ethyl 2-cyanoethyl N,N-diisopropylphosphoramidite *(**1**)*

*N*-benzyl-2,2,2-trifluoro-*N*-(2-(2-hydroxyethoxy)ethyl)acetamide (**3**, 670 mg, 2.30 mmol) and Et_3_N (1.93 mL, 13.8 mmol) were dissolved in anhydrous CH_2_Cl_2_ (7 mL) under N_2_ atmosphere. 2-Cyanoethyl *N*,*N*-diisopropylchlorophosphoramidite (0.616 mL, 2.76 mmol) was added and the resulting mixture stirred for 3 h at 25 °C, after which the reaction was quenched by addition of saturated aq. NaHCO_3_ (100 mL). The phases were separated and the aqueous phase was extracted with CH_2_Cl_2_ (100 mL). The combined organic phases were washed with saturated aq. NaHCO_3_ (100 mL), dried over anhydrous Na_2_SO_4_ and evaporated to dryness. The crude product thus obtained was passed through a silica gel column eluting with a mixture of EtOAc and hexane (1:1, *v*/*v*) to afford the desired product **1** containing a major impurity of 2-Cyanoethyl *N*,*N*-diisopropylphosphonamidate (859 mg, 72% yield based on ^31^P NMR). This material was used in oligonucleotide synthesis without further purification. ^1^H NMR (CDCl_3_, 500 MHz, major rotamer): δ 7.22-7.48 (m, 5H), 4.80 (s, 2H), 3.36-3.89 (m, 12H), 2.66 (m, 2H), 1.20 (d, *J* = 6.8 Hz, 6H), 1.19 (d, *J* = 6.8 Hz, 6H). ^1^H NMR (CDCl_3_, 500 MHz, minor rotamer): δ 7.22-7.48 (m, 5H), 4.82 (s, 2H), 3.36-3.89 (m, 12H), 2.66 (m, 2H), 1.20 (d, *J* = 6.8 Hz, 6H), 1.19 (d, *J* = 6.8 Hz, 6H). ^13^C NMR (CDCl_3_, 125 MHz, major rotamer): δ 157.1 (q, *J* = 36.0 Hz), 136.3, 128.7, 127.7, 127.6, 118.6, 116.8 (q, *J* = 288.2 Hz), 71.1 (d, *J* = 7.6 Hz), 67.8, 62.6 (d, *J* = 16.8 Hz), 58.4 (d, *J* = 19.2 Hz), 50.3, 46.7 (q, *J* = 2.9 Hz), 42.9 (d, *J* = 12.6 Hz), 24.0 (d, *J* = 7.2 Hz), 20.0. ^13^C NMR (CDCl_3_, 125 MHz, minor rotamer): δ 156.7 (q, *J* = 35.4 Hz), 135.7, 129.0, 127.9, 127.2, 118.0, 116.8 (q, *J* = 288.7 Hz), 71.0 (d, *J* = 7.7 Hz), 69.1, 62.7 (d, *J* = 17.1 Hz), 58.4 (d, *J* = 19.2 Hz), 51.5 (q, *J* = 3.2 Hz), 45.9, 42.9 (d, *J* = 12.6 Hz), 24.0 (d, *J* = 7.2 Hz), 20.0 (d, *J* = 6.9 Hz). ^31^P NMR (CDCl_3_, 202 MHz, major rotamer): δ 148.1. ^31^P NMR (CDCl_3_, 202 MHz, minor rotamer): δ 148.0. HRMS (ESI^+^) *m*/*z* calcd 514.2053 obsd 514.2034 [M + Na]^+^.

### 4.4. Bis{N-[2-(2-hydroxyethoxy)ethyl]benzylaminato-C^2^,N} bis(µ-chloro) dipalladium(II) *(**4**)*

2-[2-(benzylamino)ethoxy]ethanol (**2**, 240 mg, 1.28 mmol) was dissolved in a mixture of H_2_O (3 mL) and MeCN (3 mL). Li_2_PdCl_4_ (336 mg, 1.28 mmol) was dissolved in a mixture of H_2_O (10 mL) and MeCN (10 mL) and the resulting solution was added to the solution of **2**. After stirring for 16 h at 25 °C, the reaction mixture was evaporated to dryness. The residue was purified by silica gel column chromatography eluting with a mixture of EtOAc and hexane (8:2, *v*/*v*), affording the desired product **4** as a mixture of *cisoid* and *transoid* stereoisomers (189 mg, 44% yield). ^1^H NMR (CDCl_3_, 500 MHz, major stereoisomer): δ 7.34-7.47 (m, 4H), 4.46 (ddd, *J*_1_ = 10.6 Hz, *J*_2_ = 10.4 Hz, *J*_3_ = 2.5 Hz, 1H), 4.28 (dd, *J*_1_ = 13.1 Hz, *J*_2_ = 6.7 Hz, 1H), 4.03 (m, 1H), 3.44-3.80 (m, 6 H), 3.02 (m, 1H), 2.38 (m, 1H). ^1^H NMR (CDCl_3_, 500 MHz, minor stereoisomer): δ 7.34-7.47 (m, 4H), 4.33 (m, 1H), 4.24 (dd, *J*_1_ = 13.0 Hz, *J*_2_ = 7.6 Hz, 1H), 4.03 (m, 1H), 3.44-3.80 (m, 6 H), 3.02 (m, 1H), 2.38 (m, 1H). ^13^C NMR (CDCl_3_, 125 MHz, major stereoisomer): δ 147.5, 135.0, 129.9, 129.7, 128.9, 128.6, 72.8, 67.8, 61.6, 57.35, 50.8. ^13^C NMR (CDCl_3_, 125 MHz, minor stereoisomer): δ 142.7, 135.2, 129.9, 129.7, 128.9, 128.6, 72.8, 67.6, 61.4, 57.26, 51.5. HRMS (ESI^+^) *m/z* calcd 300.0216 obsd 300.0152 [M/2 − Cl]^+^.

### 4.5. Oligonucleotide Synthesis

The modified oligonucleotides **ON1b**, **ON2b**, **ON3b** and **ON4b** were assembled on an Applied Biosystems 3400 (Applied Biosystems, Waltham, MA, USA) automated DNA/RNA synthesizer using conventional phosphoramidite strategy. For the benzylamine building block **1**, an extended coupling time (600 s) was used. Removal of the base and phosphate protections and release of the oligonucleotides from the solid support was accomplished by treatment with 25% aq. NH_3_ for 16 h at 55 °C. The cyclopalladated oligonucleotides **ON1b-Pd**, **ON2b-Pd**, **ON3b-Pd** and **ON4b-Pd** were prepared by incubating **ON1b**, **ON2b**, **ON3b** and **ON4b** (192, 260, 290 and 173 nmol, respectively) and Li_2_PdCl_4_ (384, 520, 580 and 346 nmol, respectively) in a mixture of H_2_O (530 µL) and MeCN (30 µL) for 16 h at 25 °C. All modified oligonucleotides were purified by reversed-phase high performance liquid chromatography (RP-HPLC) on a Hypersil ODS C18 column (250 × 4.6 mm, 5 µm, Thermo Fisher Scientific, Waltham, MA, USA) eluting with a linear gradient (0 to 30% over 25 min) of MeCN in 50 mM aqueous triethylammonium acetate. The flow rate was 1.0 mL·min^−1^ and the detection wavelength 260 nm. The purified oligonucleotides were characterized by electrospray ionization mass spectrometry (ESI-MS) and quantified UV spectrophotometrically using molar absorptivities calculated by an implementation of the nearest-neighbors method. Molar absorptivity of both free and cyclopalladated benzylamine was assumed to be negligible.

### 4.6. Melting Temperature Measurements

Melting profiles were recorded on a PerkinElmer Lambda 35 UV-Vis spectrometer equipped with a Peltier temperature control unit (PerkinElmer, Waltham, MA, USA). Samples were prepared by mixing the appropriate oligonucleotides (3.0 µM) in 20 mM cacodylate buffer (pH 7.4), the ionic strength of which was adjusted to 0.10 M with NaClO_4_. When applicable, 2-mercaptoethanol was used in 100 µM concentration and added after mixing of the oligonucleotides. Before measurement, the samples were annealed by heating to 90 °C and gradually cooling to room temperature. UV melting curves were acquired by monitoring the absorbance at λ = 260 nm over a temperature range of 10–90 °C, sampling at 10 °C intervals. The melting temperatures were determined as inflection points on the UV melting curves.

### 4.7. CD Measurements

CD spectra were recorded on an Applied Photophysics Chirascan spectropolarimeter equipped with a Peltier temperature control unit (Applied Photophysics, Leatherhead, UK). Samples used in the CD measurements were identical to those used in the UV melting temperature measurements. CD spectra were acquired between λ = 200 and 400 nm over a temperature range of 10–90 °C, sampling at 10 °C intervals. At each temperature, samples were allowed to equilibrate for 600 s before acquisition.

## 5. Conclusions

Cyclopalladated benzylamine, tethered at a terminal position of a short oligonucleotide by a flexible linker, promotes hybridization despite a significant entropy penalty for “freezing” the linker in an appropriate conformation. Sensitivity to the presence of competing ligands suggests direct coordination of Pd(II) to a nucleobase of the complementary strand as the origin of stabilization by the palladacyclic moiety. In light of these results, oligonucleotides furnished with palladacyclic “warheads” could prove useful as a sequence-selective alternative to current platinum-based anticancer agents.

## Figures and Tables

**Table 1:** Oligonucleotides used in this study.

Sequence ^1^	Oligonucleotide
**ON1a**	5′-AGCTCTGGC-3′
**ON2a**	5′-AGCTCTGG-3′
**ON3a**	5′-AGCTCTG-3′
**ON4a**	5′-AGCTCT-3′
**ON1b**	5′-BGCTCTGGC-3′
**ON2b**	5′-BGCTCTGG-3′
**ON3b**	5′-BGCTCTG-3′
**ON4b**	5′-BGCTCT-3′
**ON1b-Pd**	5′-B^Pd^GCTCTGGC-3′
**ON2b-Pd**	5′-B^Pd^GCTCTGG-3′
**ON3b-Pd**	5′-B^Pd^GCTCTG-3′
**ON4b-Pd**	5′-B^Pd^GCTCT-3′
**ON5a**	5′-GCCAGAGCTCG-3′
**ON5c**	5′-GCCAGCGCTCG-3′
**ON5g**	5′-GCCAGGGCTCG-3′
**ON5t**	5′-GCCAGTGCTCG-3′

^1^ B refers to unmetalated and B^Pd^ to cyclopalladated benzylamine residue. The residues varied in the hybridization studies have been underlined.

**Table 2:** Thermodynamic parameters of hybridization for **ON1a**•**ON5a**, **ON1b**•**ON5a** and **ON1b-Pd**•**ON5a**; pH = 7.4 (20 mM cacodylate buffer); [oligonucleotides] = 3.0 µM; *I*(NaClO_4_) = 0.10 M.

Duplex	Δ*H*°/kJ·mol^−1^	Δ*S*°/J·mol^−1^·K^−1^
**ON1a**•**ON5a**	−200 ± 20	−530 ± 50
**ON1b**•**ON5a**	−200 ± 10	−550 ± 40
**ON1b-Pd**•**ON5a**	−250 ± 20	−670 ± 50

## Supplementary Materials

Supplementary materials can be found at http://www.mdpi.com/1422-0067/19/6/1588/s1.

## Abbreviations

CD	circular dichroism
DNA	deoxyribonucleic acid
PNA	peptide nucleic acid
RP-HPLC	reversed-phase high performance liquid chromatography
ESI-MS	electrospray ionization mass spectrometry
