# Supplementary material for: Palladacyclic Conjugate Group Promotes Hybridization of Short Oligonucleotides
Source: Int J Mol Sci. 2018 May 28;19(6):1588. doi: 10.3390/ijms19061588 (PMC6032164; doi:10.3390/ijms19061588)
Supplement: Supplementary file 1 [file ijms-19-01588-s001.pdf]

Supporting Information for

Palladacyclic conjugate group promotes hybridization of short oligonucleotides

Madhuri Ashok Hande, Sajal Kumar Maity and Tuomas Lönnberg*

Department of Chemistry, University of Turku

Email: tuanlo@utu.fi

Contents

^{1}H NMR spectrum of compound 3 S2
^{13}C NMR spectrum of compound 3 S3
^{1}H NMR spectrum of compound 1 S4
^{13}C NMR spectrum of compound 1 S5
^{31}P NMR spectrum of compound 1 S7
^{1}H NMR spectrum of compound 4 S8
^{13}C NMR spectrum of compound 4 S9
Figure S1. IE-HPLC trace of oligonucleotide ON1b S11
Figure S2. IE-HPLC trace of oligonucleotide ON2b S11
Figure S3. IE-HPLC trace of oligonucleotide ON3b S12
Figure S4. IE-HPLC trace of oligonucleotide ON4b S12
Figure S5. RP-HPLC traces of oligonucleotides ON1b-Pd, ON2b-Pd, ON3b-Pd and ON4b-Pd S13
Figure S6. Mass spectrum of oligonucleotide ON1b S14
Figure S7. Mass spectrum of oligonucleotide ON2b S14
Figure S8. Mass spectrum of oligonucleotide ON3b S15
Figure S9. Mass spectrum of oligonucleotide ON4b S15
Figure S10. Mass spectrum of oligonucleotide ON1b-Pd S16
Figure S11. Mass spectrum of oligonucleotide ON2b-Pd S16
Figure S12. Mass spectrum of oligonucleotide ON3b-Pd S17
Figure S13. Mass spectrum of oligonucleotide ON4b-Pd S17
Table S1. Melting temperatures of various duplexes S18
Table S2. Melting temperatures of various duplexes in the presence of 2-mercaptoethanol S18
Figure S14. CD spectra of ON1b-Pd•ON5a S19
Figure S15. CD spectra of ON1b-Pd•ON5c S19
Figure S16. CD spectra of ON1b-Pd•ON5g S20
Figure S17. CD spectra of ON1b-Pd•ON5t S20
Figure S18. CD spectra of ON2b-Pd•ON5a S21
Figure S19. CD spectra of ON2b-Pd•ON5c S21
Figure S20. CD spectra of ON2b-Pd•ON5g S22
Figure S21. CD spectra of ON2b-Pd•ON5t S22
Figure S22. CD spectra of ON3b-Pd•ON5a S23
Figure S23. CD spectra of ON3b-Pd•ON5c S23
Figure S24. CD spectra of ON3b-Pd•ON5g S24
Figure S25. CD spectra of ON3b-Pd•ON5t S24
Figure S26. CD spectra of ON4b-Pd•ON5a S25
Figure S27. CD spectra of ON4b-Pd•ON5c S25
Figure S28. CD spectra of ON4b-Pd•ON5g S26
Figure S29. CD spectra of ON4b-Pd•ON5t S26

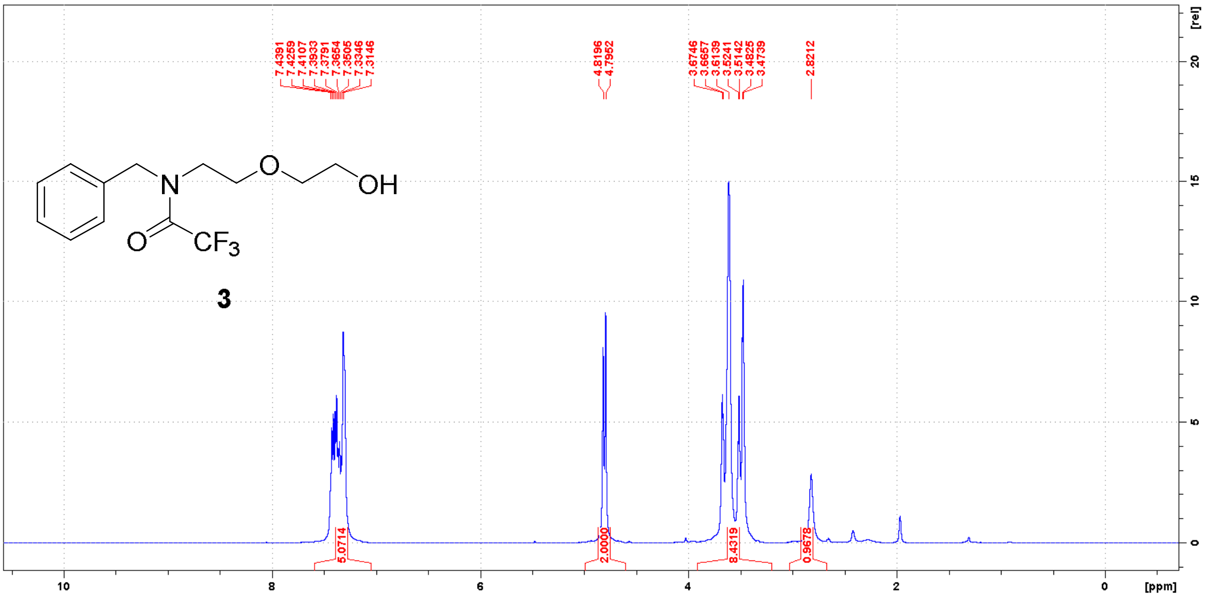
7.4391
7.4259
7.4107
7.3933
7.3791
7.3654
7.3505
7.3346
7.3146
4.8196
4.7952
3.6746
3.6657
3.6139
3.5241
3.5142
3.4825
3.4739
2.8212
OH
CF3
3
5.0714
2.0000
8.4319
0.9678
[ppm]
[rel]

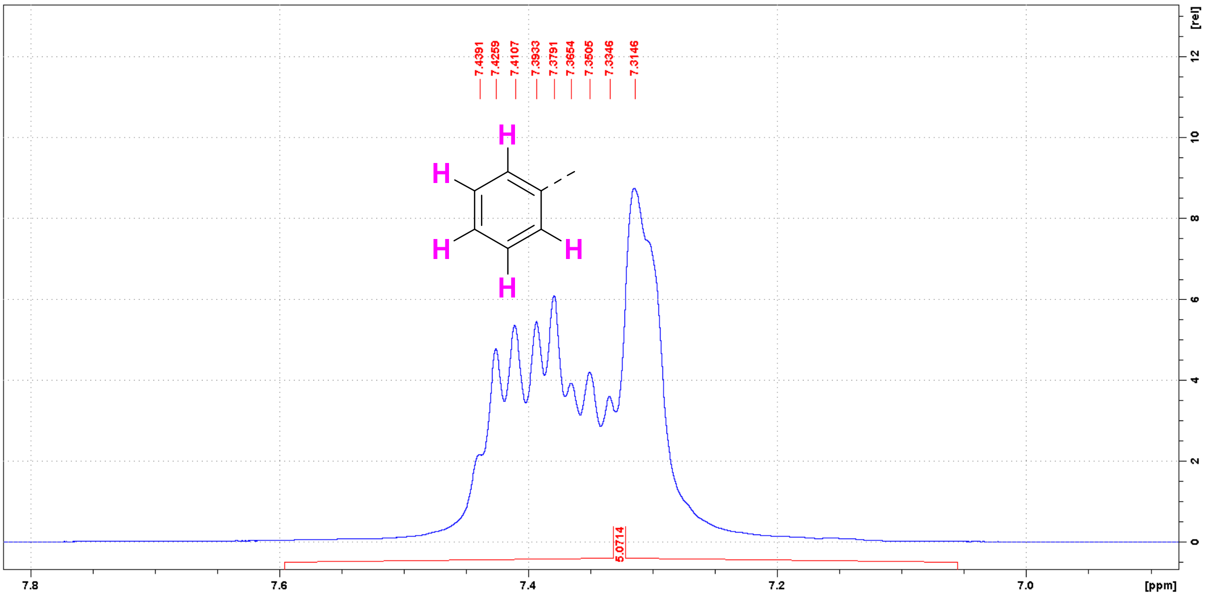
7.4391
7.4259
7.4107
7.3933
7.3791
7.3654
7.3505
7.3346
7.3146
5.0714
[ppm]
[rel]

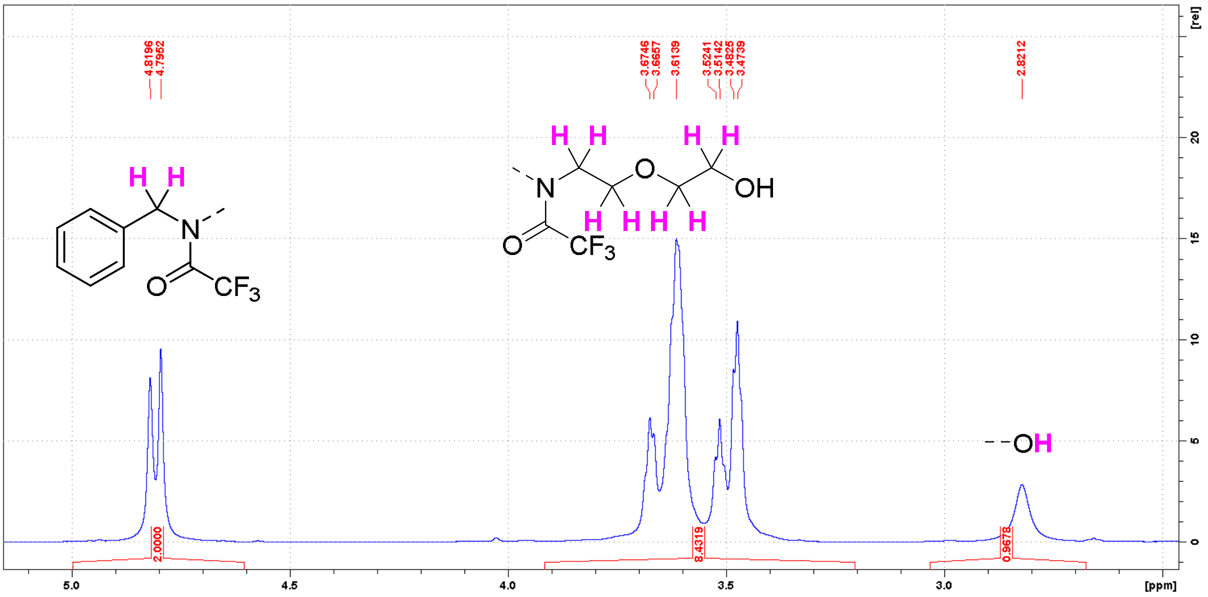
4.8196
4.7952
3.6746
3.6657
3.6139
3.5241
3.5142
3.4825
3.4739
2.8212
CF3
OH
2.0000
8.4319
0.9678
[ppm]
[rel]

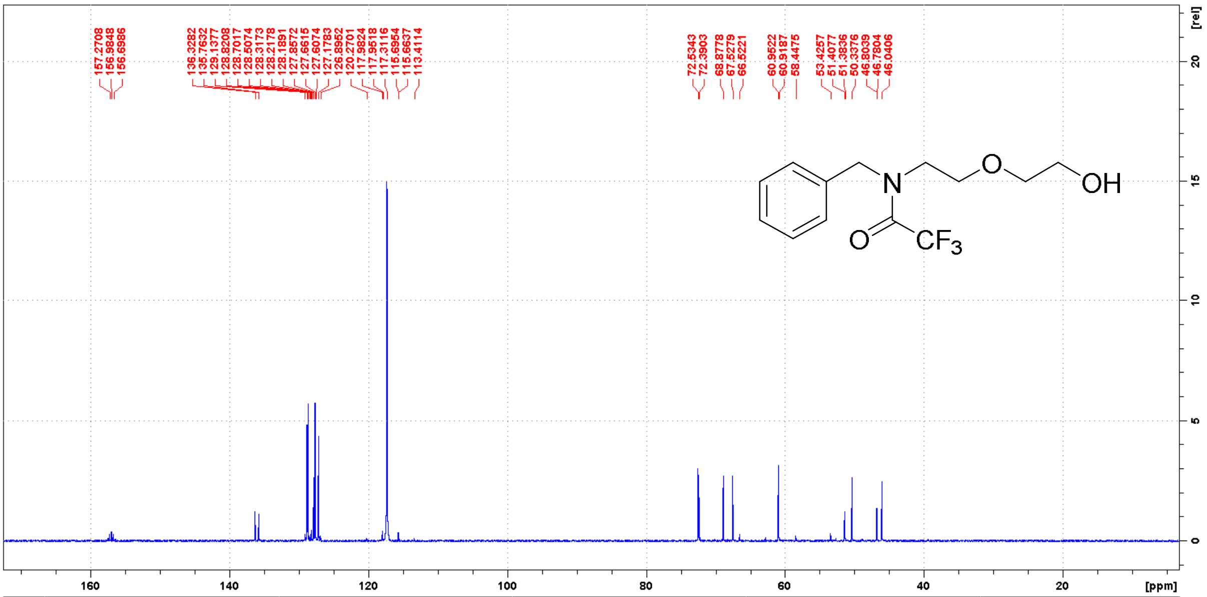
157.2708
156.9848
156.6986
136.3282
135.7632
129.1377
128.8208
128.7017
128.5074
128.3173
128.2178
128.1891
127.8572
127.6615
127.6074
127.1783
126.8952
120.2701
117.9824
117.9518
117.3116
115.6954
115.6637
113.4114
72.5343
72.3903
68.8778
67.5279
66.5221
60.9522
60.9187
58.4475
53.4257
51.4077
51.3836
50.3376
46.8039
46.7804
46.0406
N
O
OH
O
CF3
[rel]
[ppm]

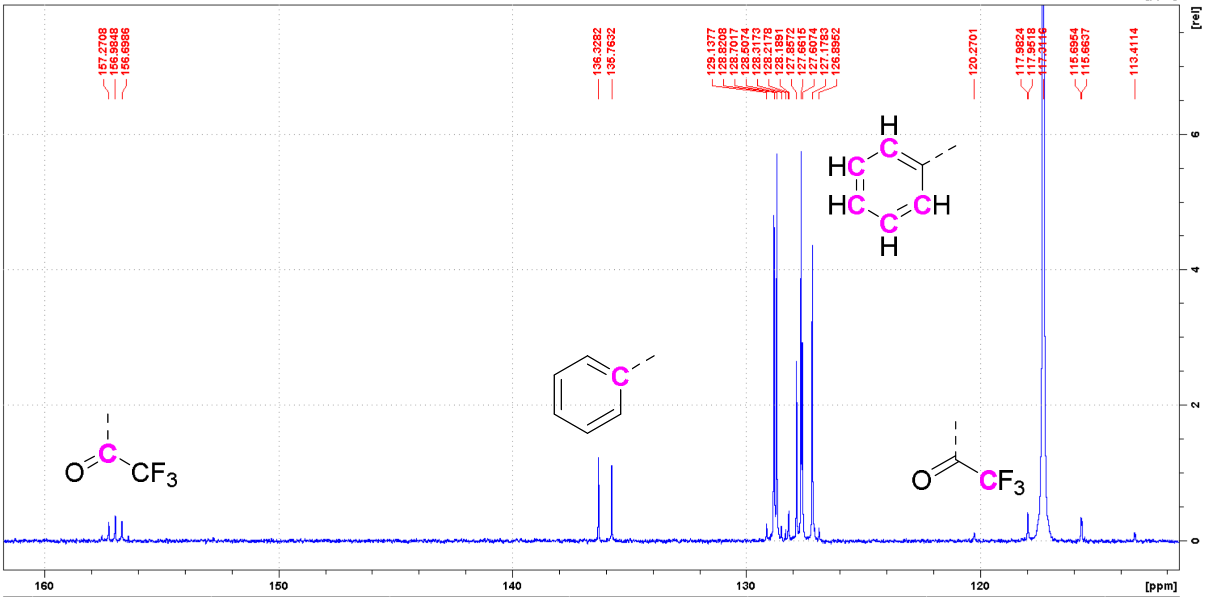
H
HC
HC
C
CH
C
H
C
O
CF3
[rel]
[ppm]

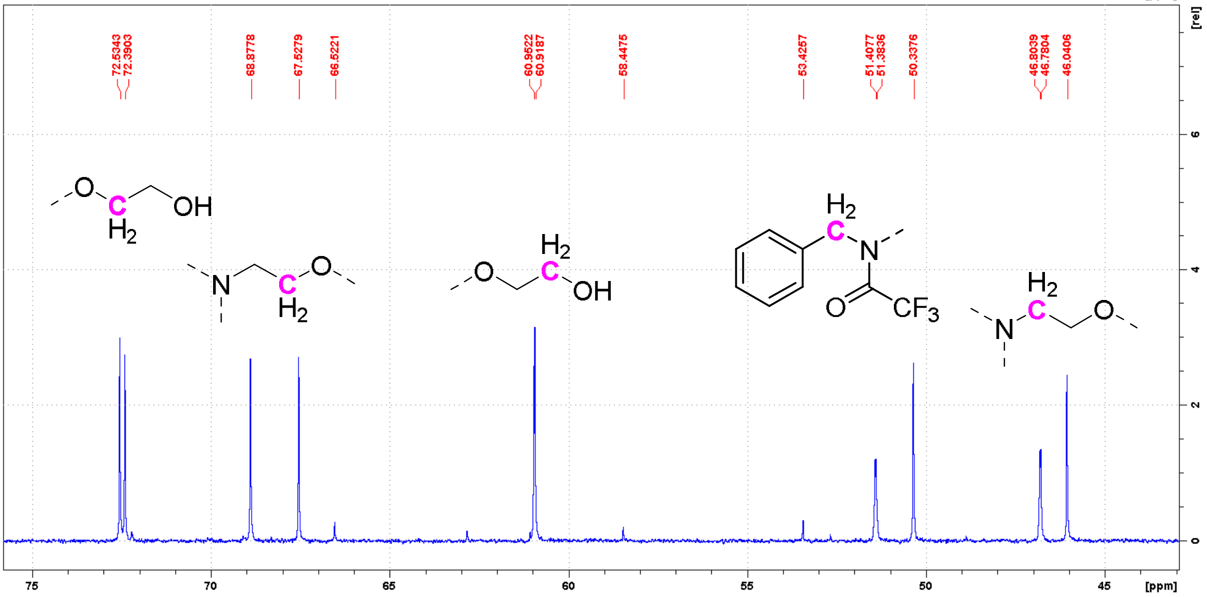
O
C
H2
OH
N
C
H2
O
H2
C
OH
N
O
CF3
[rel]
[ppm]

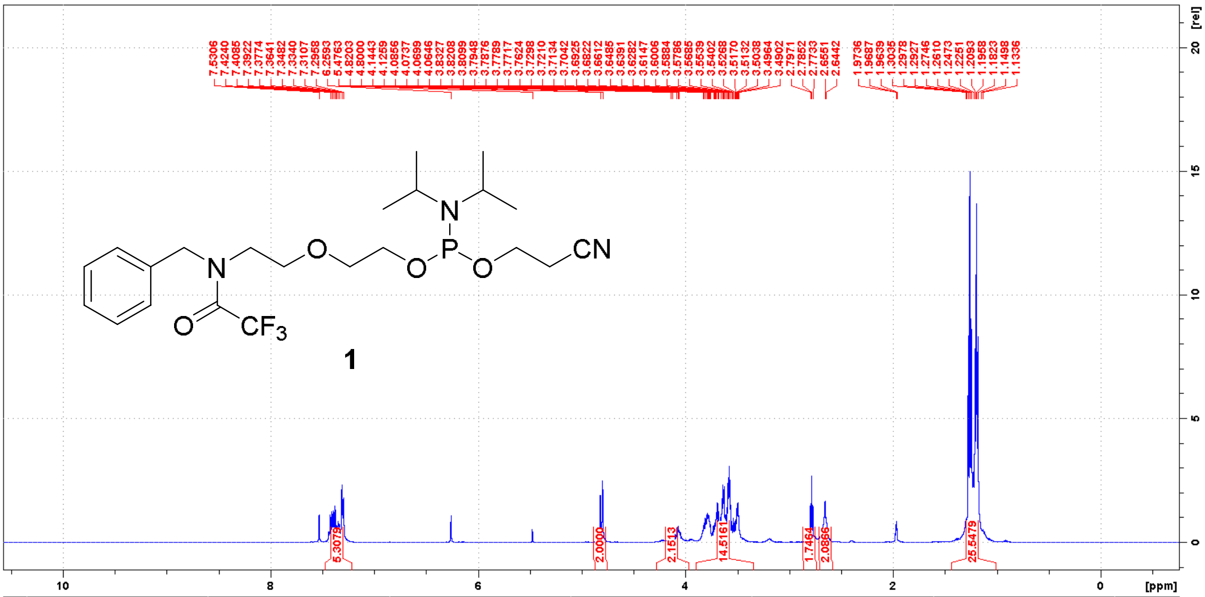

N
O
P
CN
CF3
1
[rel]
[ppm]

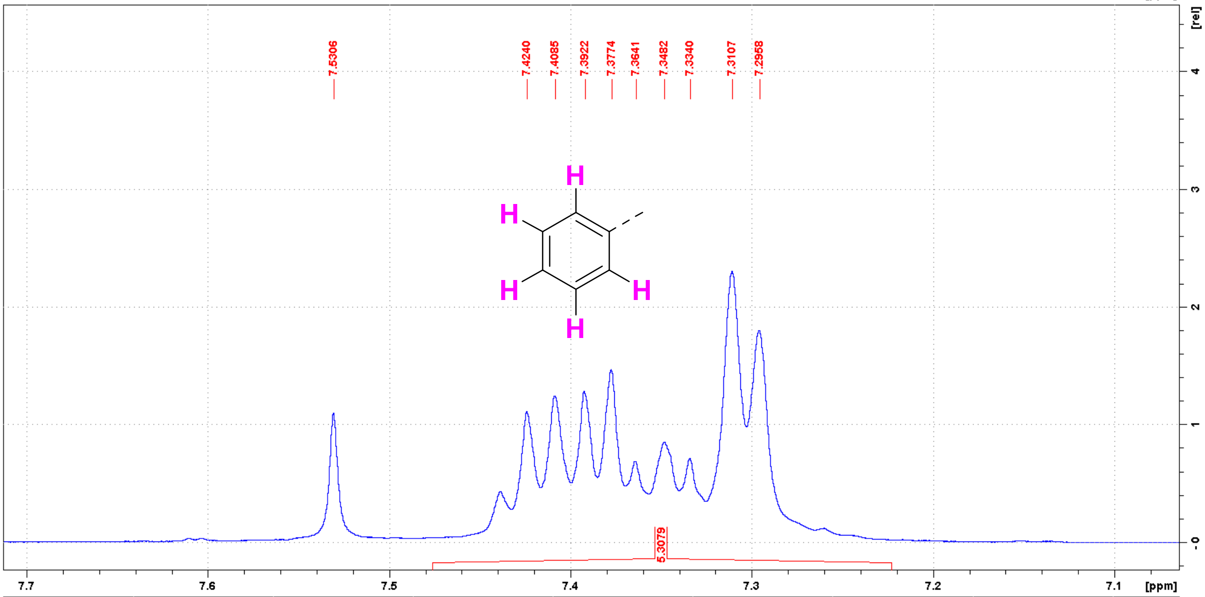

H
[rel]
[ppm]

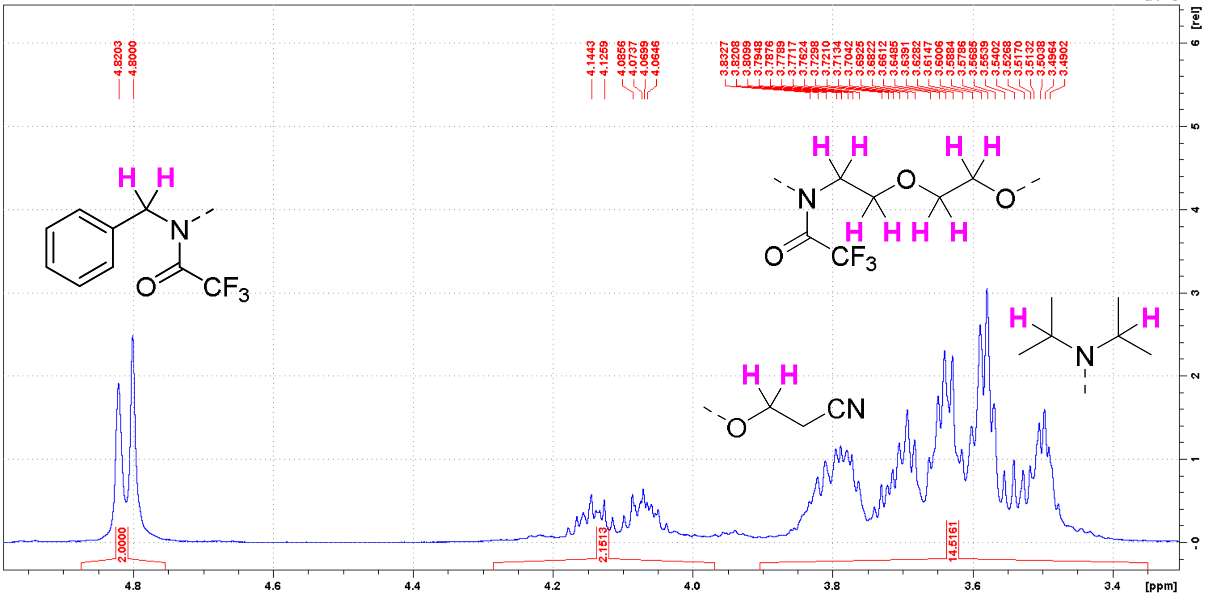

H
N
O
CF3
CN
[rel]
[ppm]

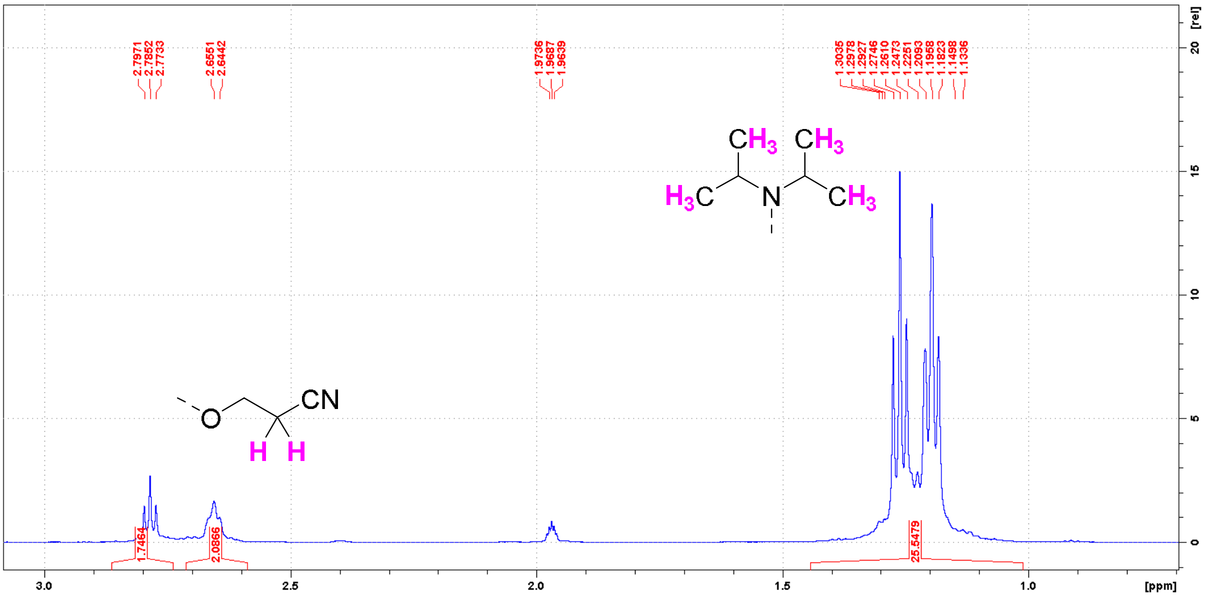
2.7971
2.7852
2.7733
2.6551
2.6442
1.9736
1.9687
1.9639
1.3035
1.2978
1.2927
1.2746
1.2610
1.2473
1.2251
1.2093
1.1958
1.1823
1.1498
1.1336
1.7464
2.0866
25.5479
CN
O
H H
CH3 CH3
H3C N CH3
[rel]
[ppm]

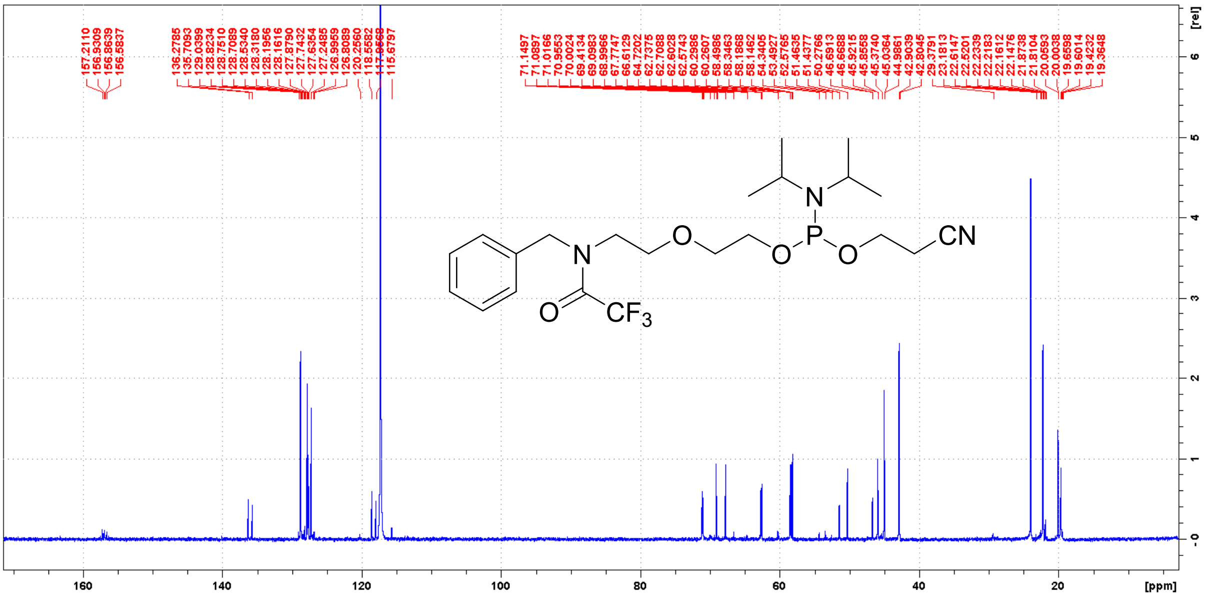
N
P
O
CN
CF3
[rel]
[ppm]

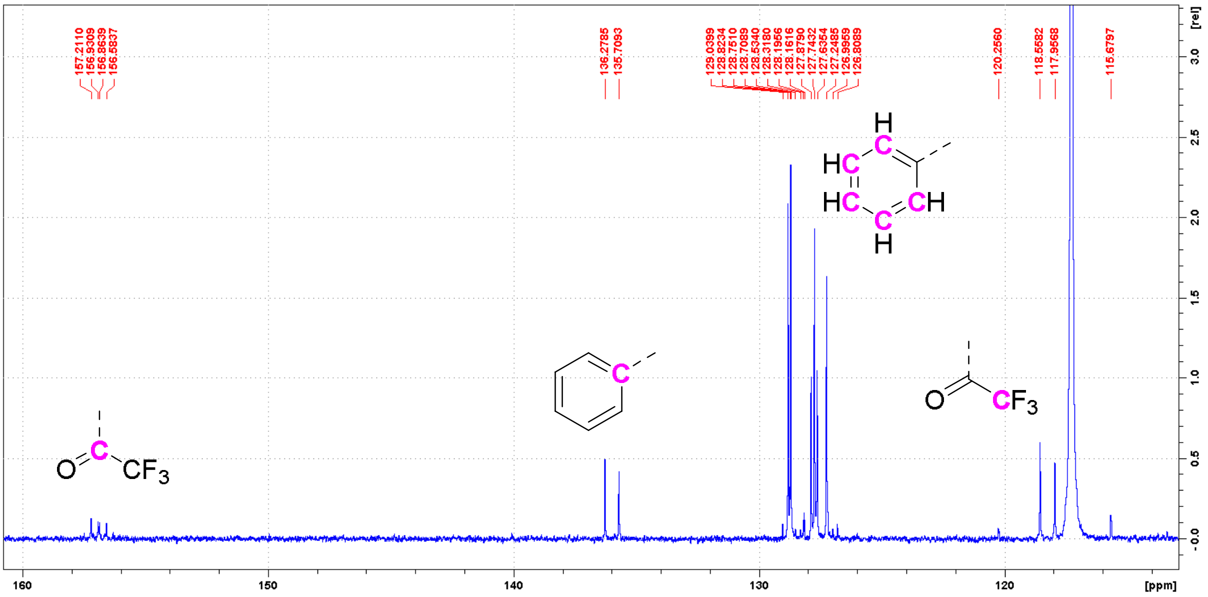
157.2110
156.9309
156.8639
156.5837
136.2785
135.7093
120.2560
118.5582
117.9568
115.6797
CF3
[rel]
[ppm]

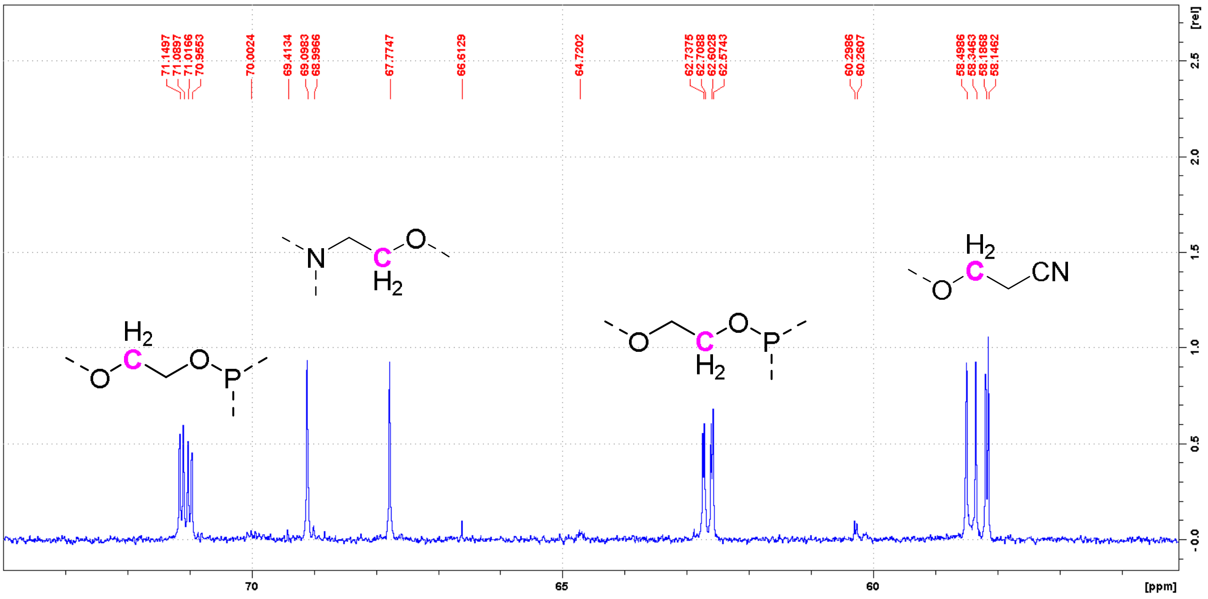
71.1497
71.0897
71.0166
70.9553
70.0024
69.4134
69.0983
68.9966
67.7747
66.6129
64.7202
62.7375
62.7088
62.6028
62.5743
60.2986
60.2607
58.4986
58.3463
58.1868
58.1462
[rel]
[ppm]

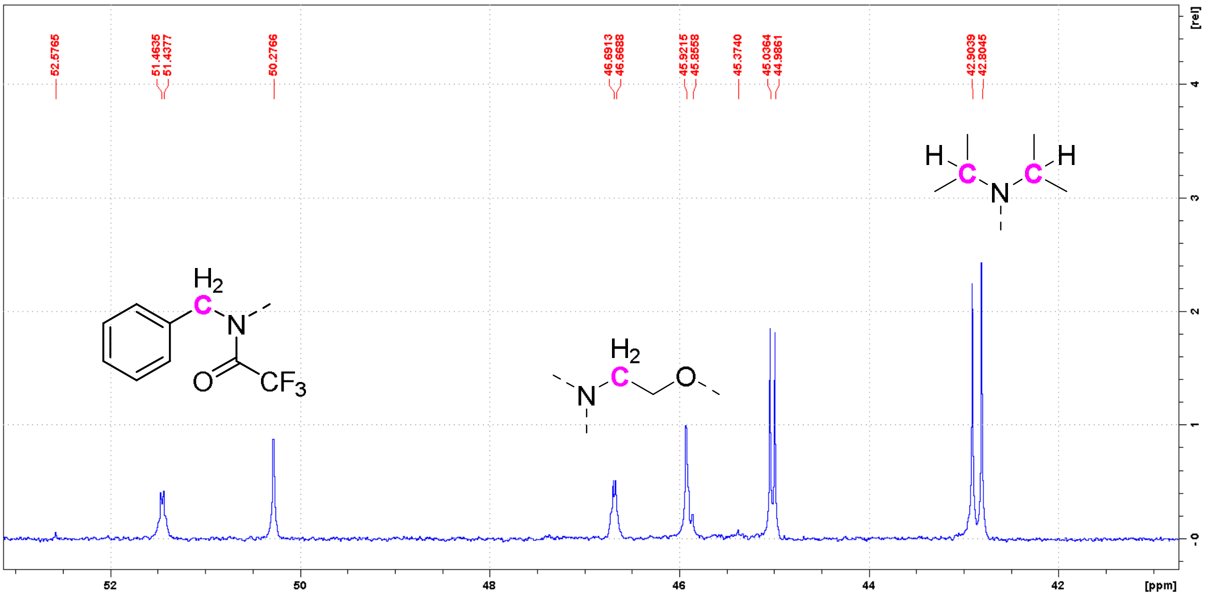
52.5765
51.4635
51.4377
50.2766
46.6913
46.6688
45.9215
45.8558
45.3740
45.0364
44.9861
42.9039
42.8045
[rel]
[ppm]

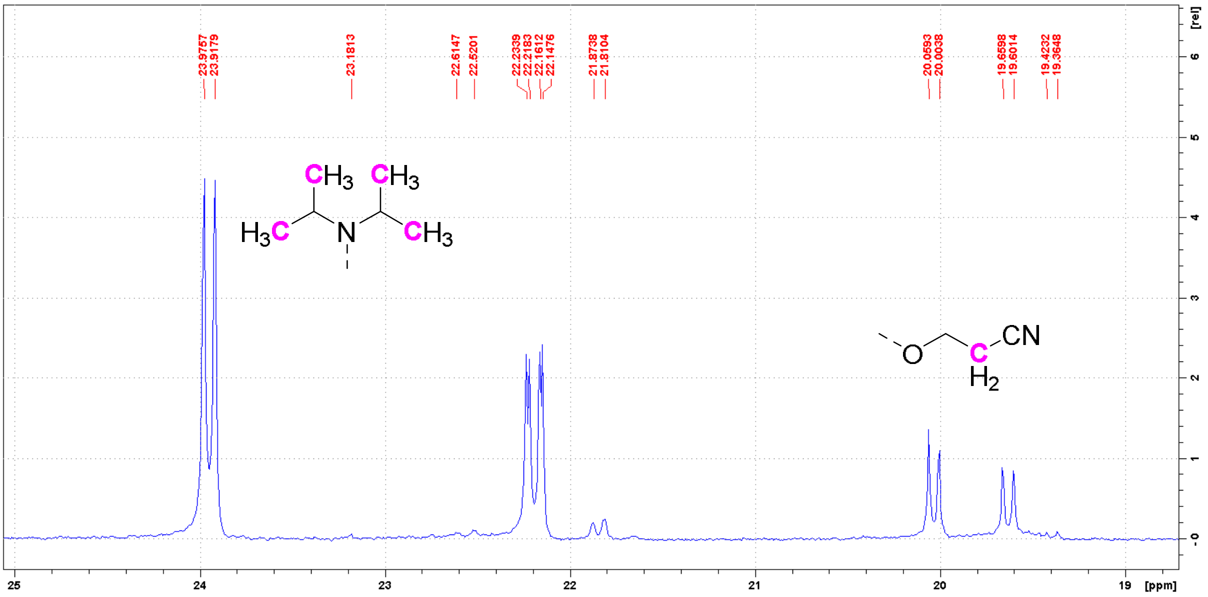
23.9757
23.9179
23.1813
22.6147
22.5201
22.2339
22.2183
22.1612
22.1476
21.8738
21.8104
20.0593
20.0038
19.6598
19.6014
19.4232
19.3648
[rel]
[ppm]

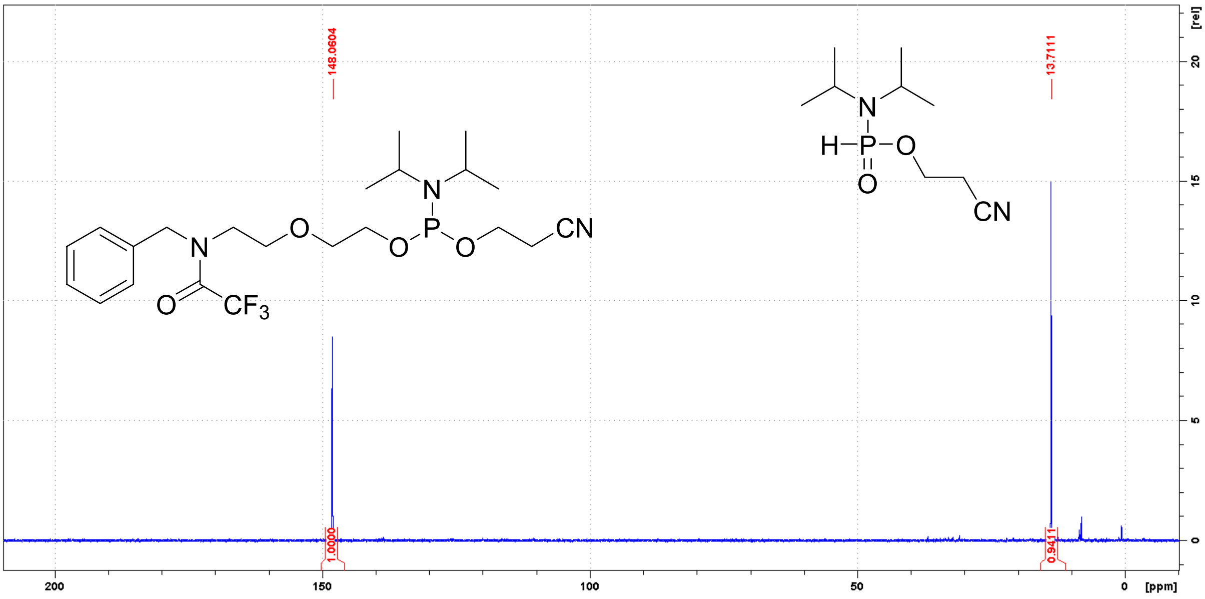

148.0604
13.7111
1.0000
0.9411
N
P
O
O
O
N
CN
O
CF3
N
H
P
O
O
CN
200
150
100
50
0
[ppm]
[rel]
20
15
10
5
0

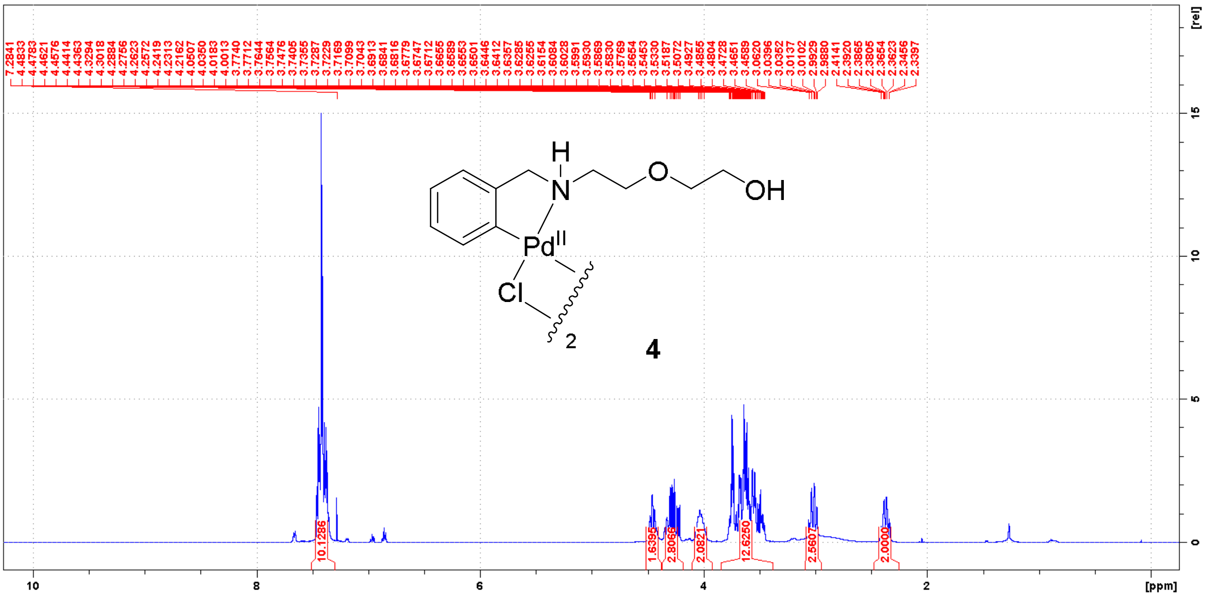

4
[ppm]
[rel]

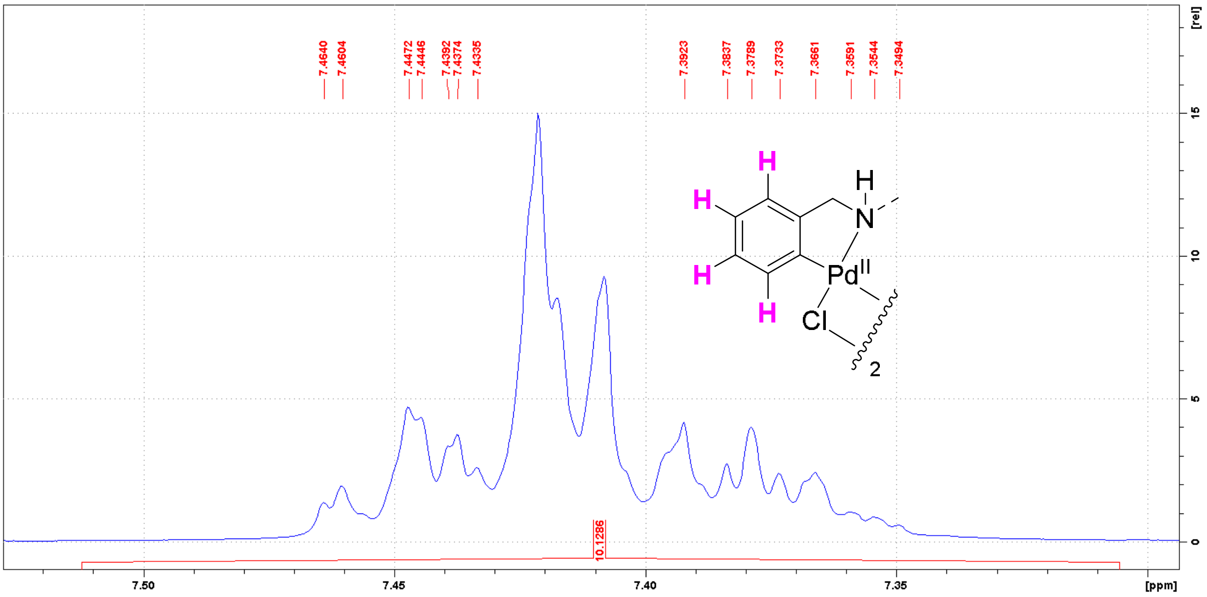

[ppm]
[rel]

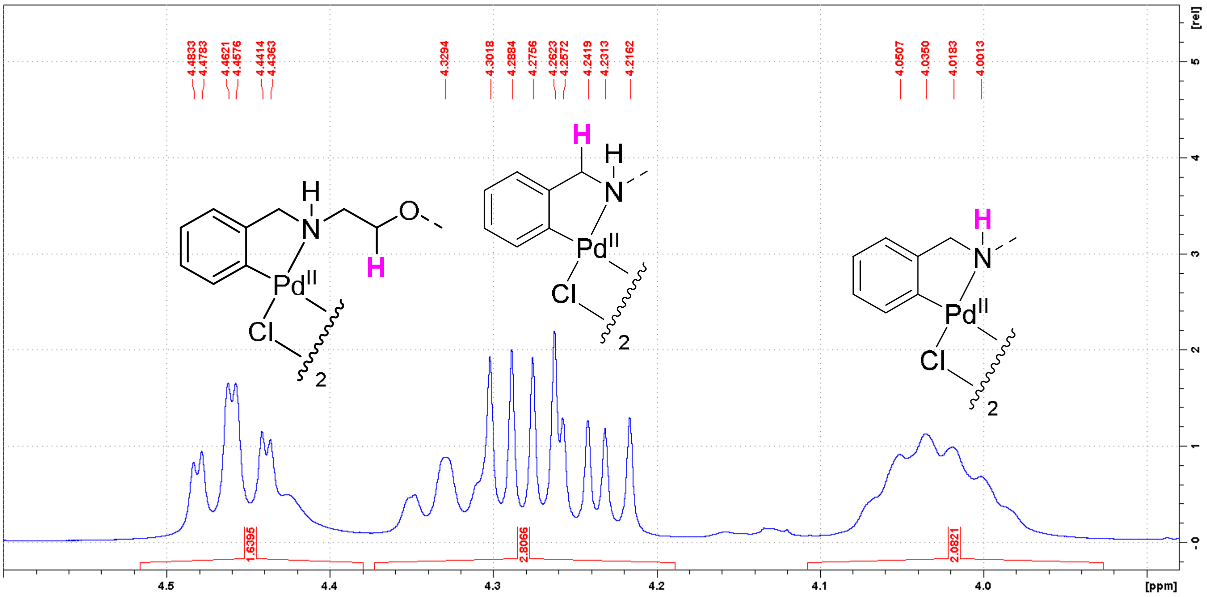

[ppm]
[rel]

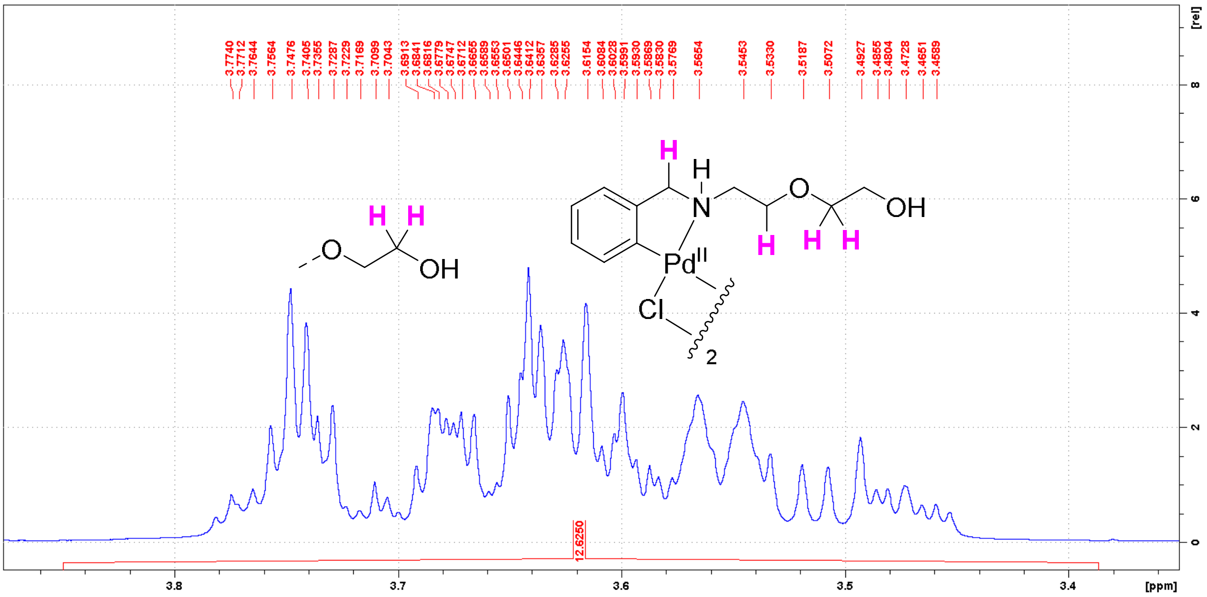
[rel]
[ppm]
3.8
3.7
3.6
3.5
3.4
12.6250
OH

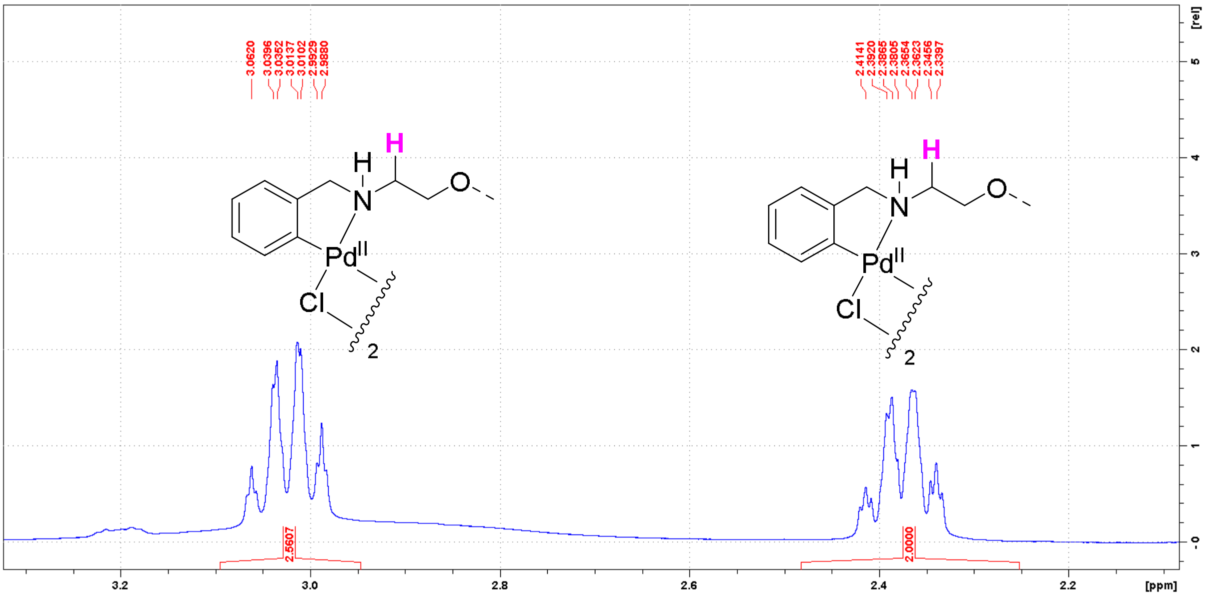
[rel]
[ppm]
3.2
3.0
2.8
2.6
2.4
2.2
2.5607
2.0000

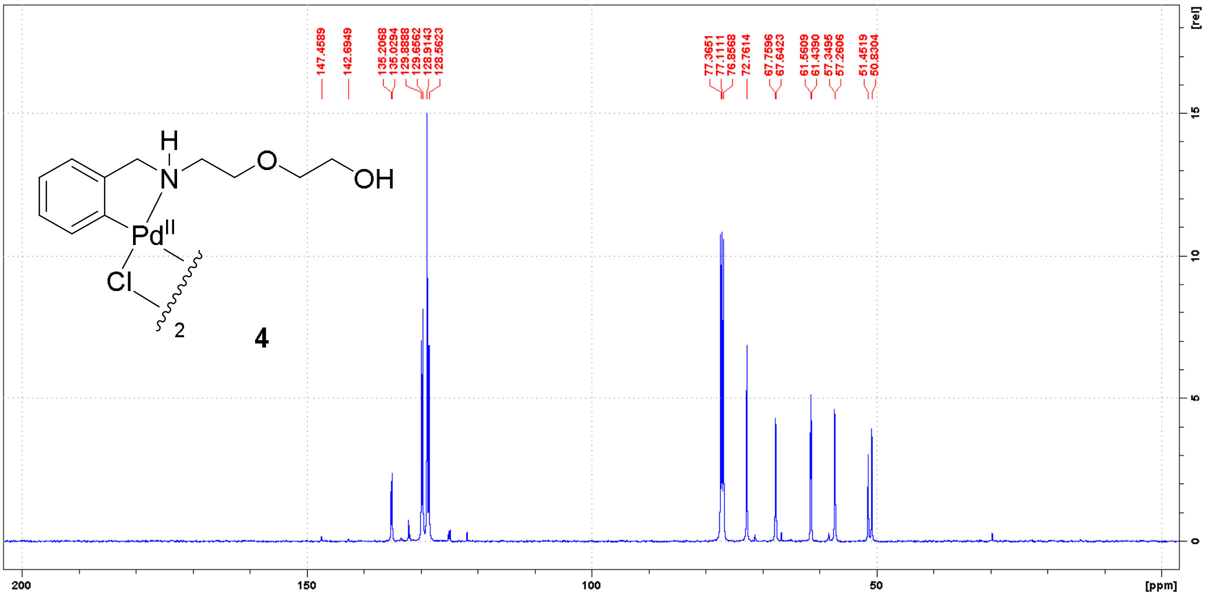
[rel]
[ppm]
200
150
100
50
OH
4

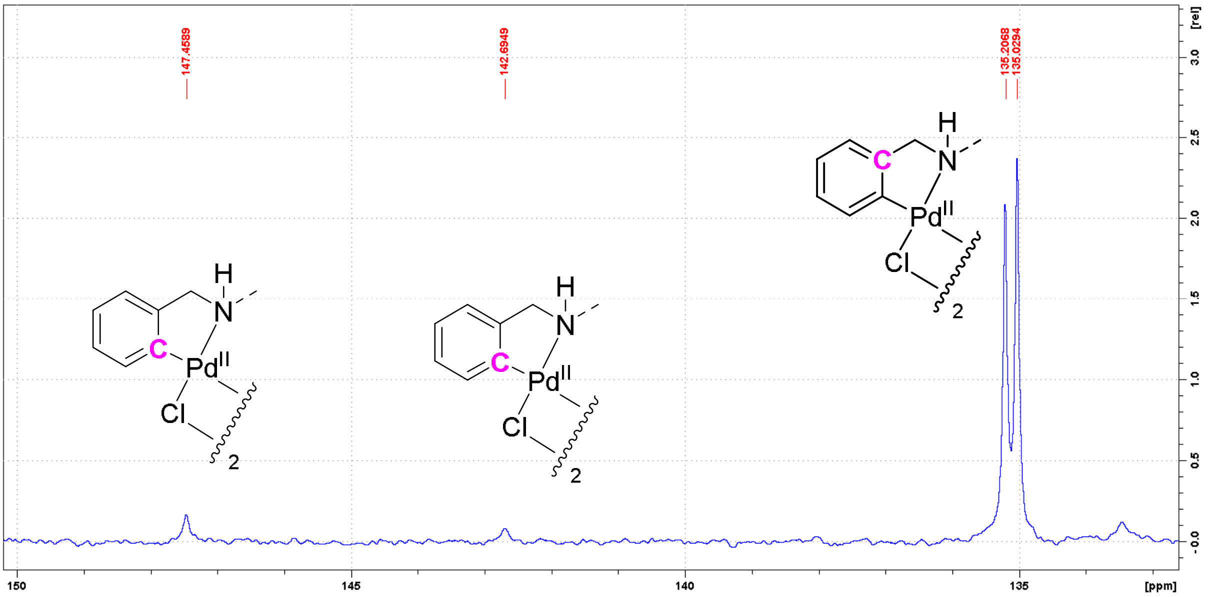
147.4589
142.6949
135.2068
135.0294
150
145
140
135
[ppm]
[rel]
H
N
C
Pd^II
Cl
2

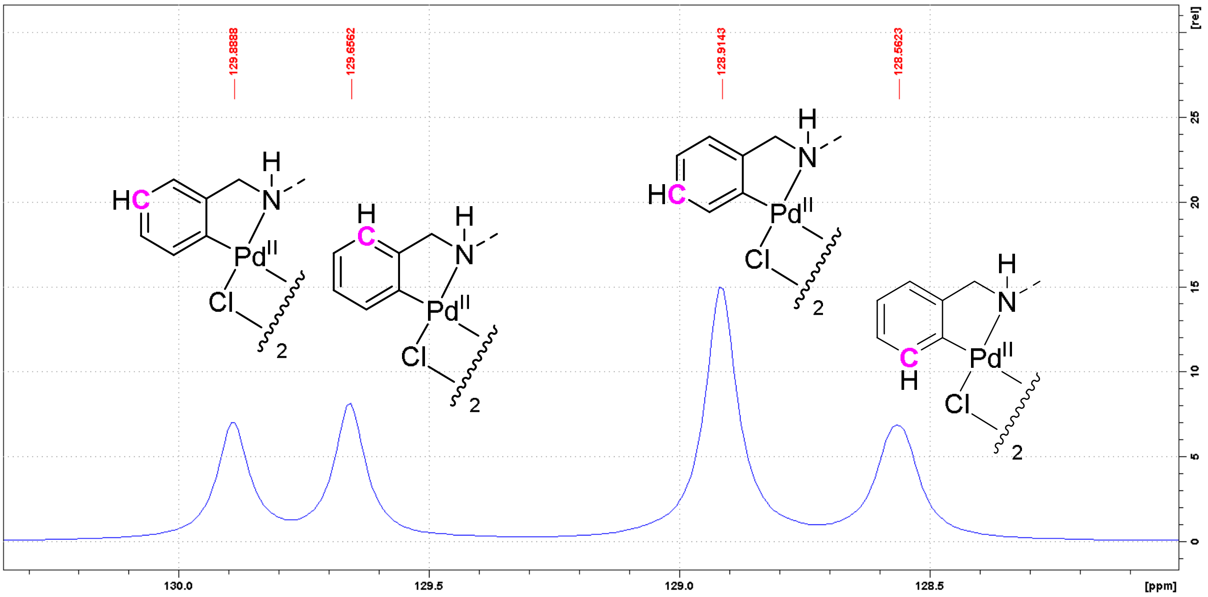
129.8888
129.6562
128.9143
128.5623
130.0
129.5
129.0
128.5
[ppm]
[rel]
HC
H
N
Pd^II
Cl
2

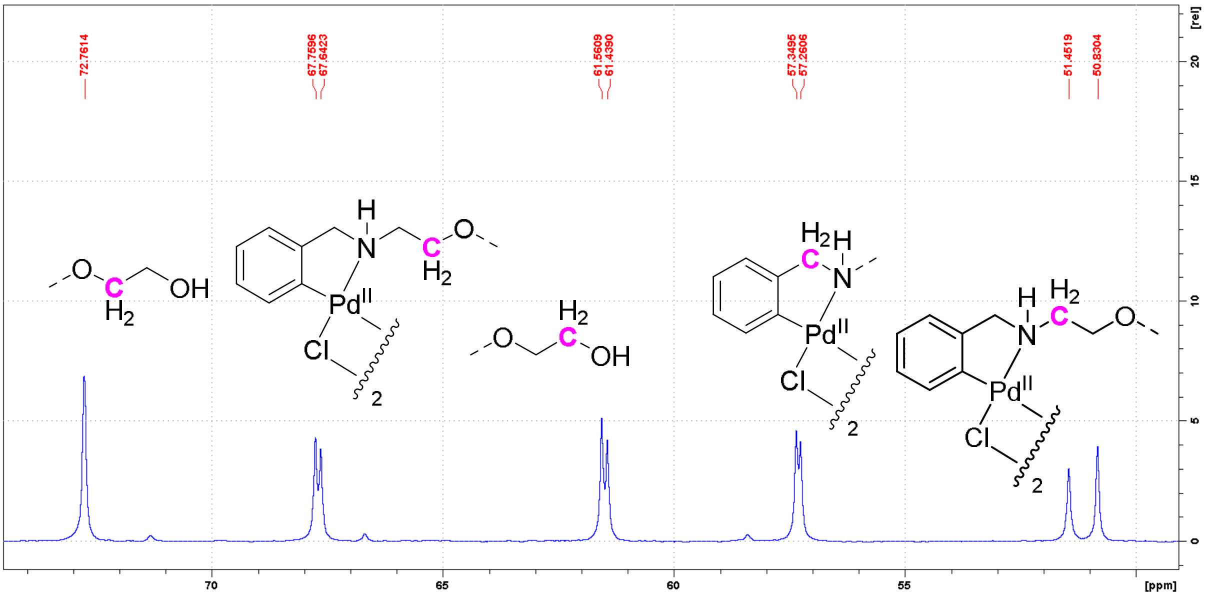
72.7614
67.7596
67.6423
61.5609
61.4390
57.3495
57.2606
51.4519
50.8304
70
65
60
55
[ppm]
[rel]
O
C
H2
OH
H
N
Pd^II
Cl
2

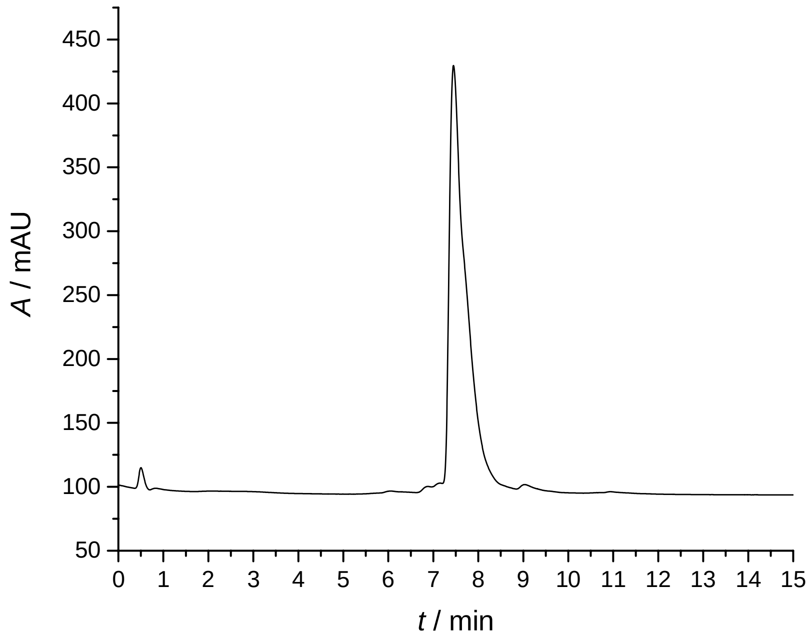

Figure S1. IE-HPLC trace of oligonucleotide ON1b; ProSwift™ SAX-1S column (50 × 1 mm, monolithic); flow rate = 0.20 mL min^{-1}; linear gradient (0.10 to 0.90 M over 15 min) of NaCl in 10 mM TRIS•HCl buffer (pH 7.6).

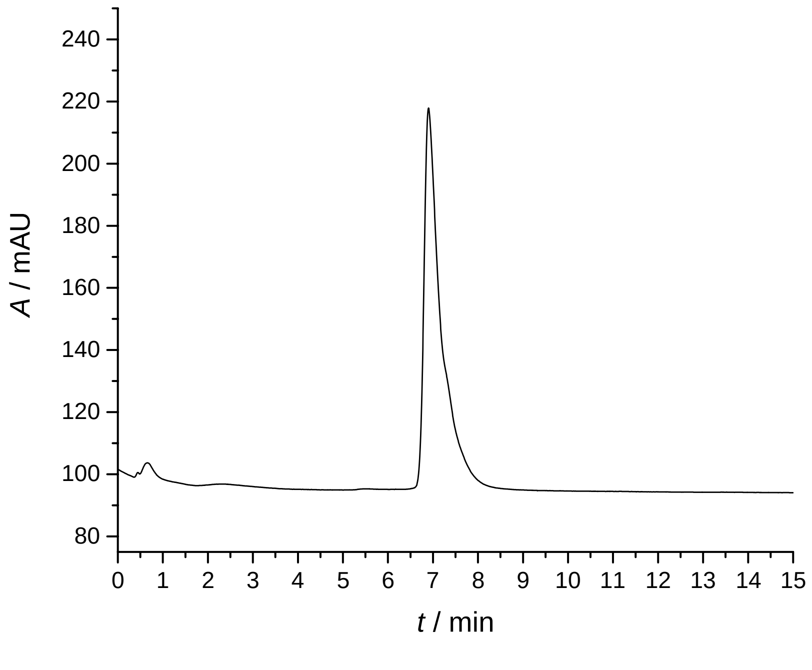

Figure S2. IE-HPLC trace of oligonucleotide ON2b; ProSwift™ SAX-1S column (50 × 1 mm, monolithic); flow rate = 0.20 mL min^{-1}; linear gradient (0.10 to 0.90 M over 15 min) of NaCl in 10 mM TRIS•HCl buffer (pH 7.6).

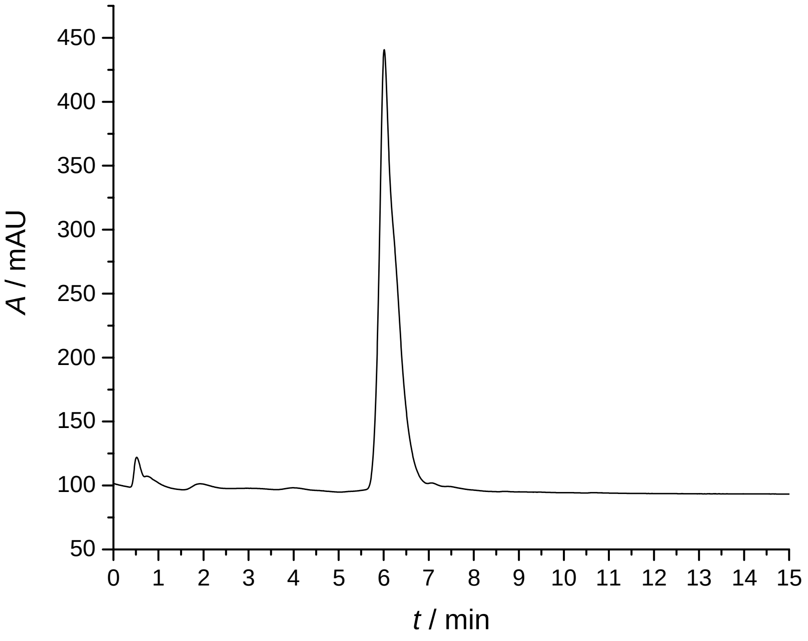

Figure S3. IE-HPLC trace of oligonucleotide ON3b; ProSwift™ SAX-1S column (50 × 1 mm, monolithic); flow rate = 0.20 mL min^{-1}; linear gradient (0.10 to 0.90 M over 15 min) of NaCl in 10 mM TRIS•HCl buffer (pH 7.6).

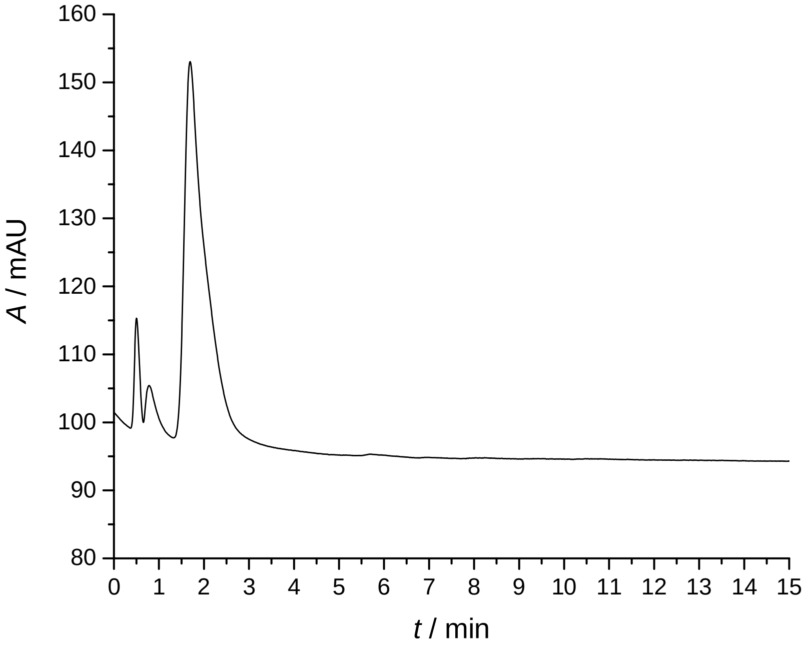

Figure S4. IE-HPLC trace of oligonucleotide ON4b; ProSwift™ SAX-1S column (50 × 1 mm, monolithic); flow rate = 0.20 mL min^{-1}; linear gradient (0.10 to 0.90 M over 15 min) of NaCl in 10 mM TRIS•HCl buffer (pH 7.6).

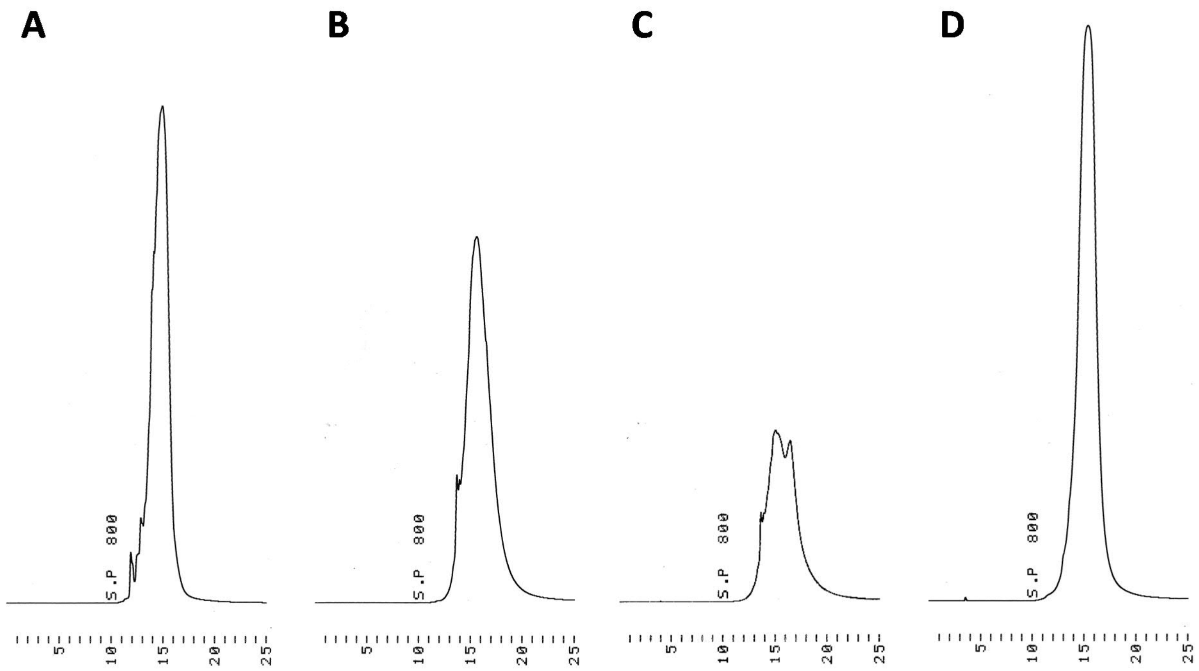

Figure S5. RP-HPLC traces of oligonucleotides A) ON1b-Pd, B) ON2b-Pd, C) ON3b-Pd and D) ON4b-Pd; Hypersil ODS C18 column (250 × 4.6 mm, 5 µm); flow rate = 1.0 mL min^{-1}; linear gradient (0 to 30% over 25 min) of MeCN in 50 mM aq. triethylammonium acetate.

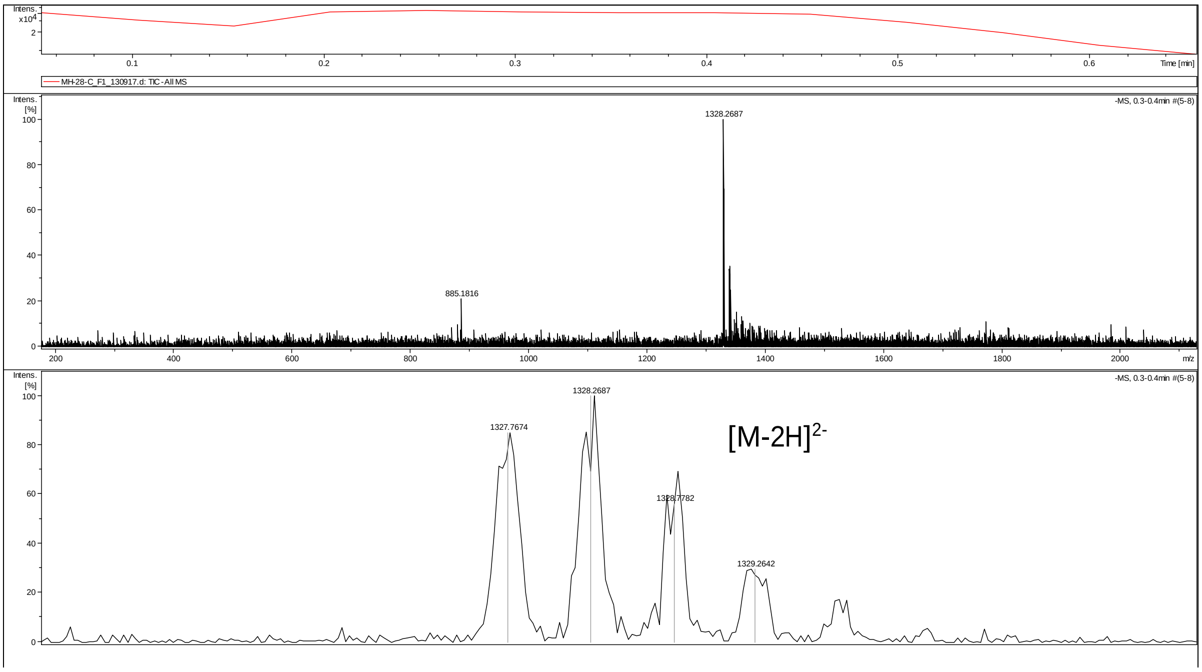

Figure S6. Mass spectrum of oligonucleotide ON1b.

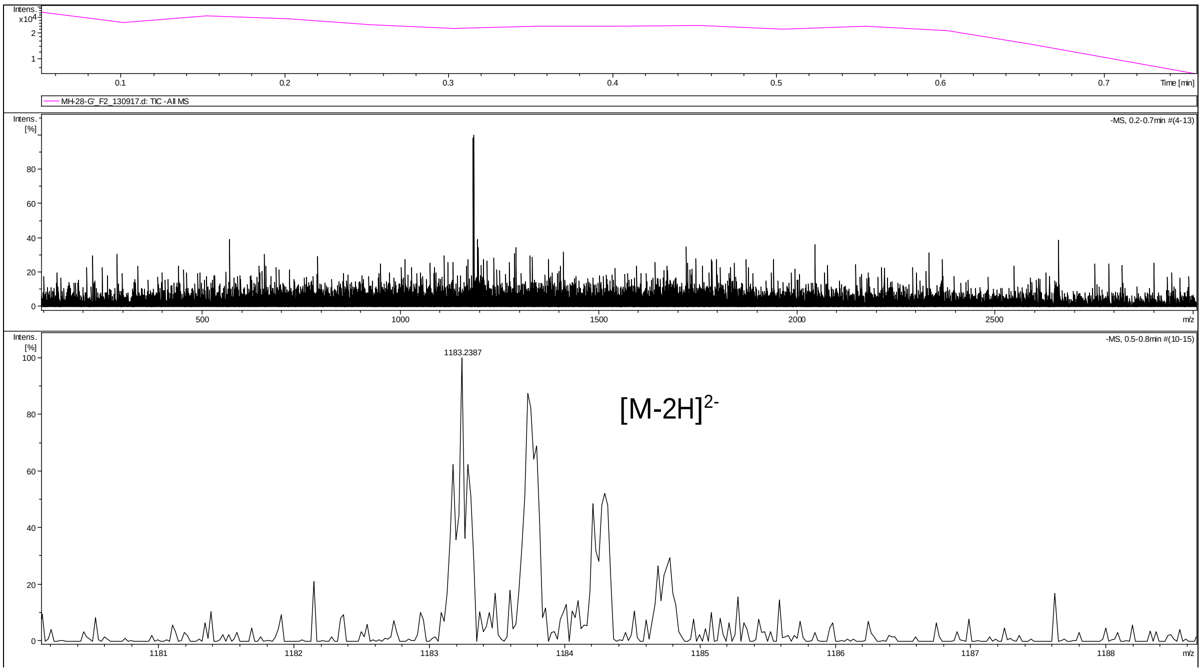

Figure S7. Mass spectrum of oligonucleotide ON2b.

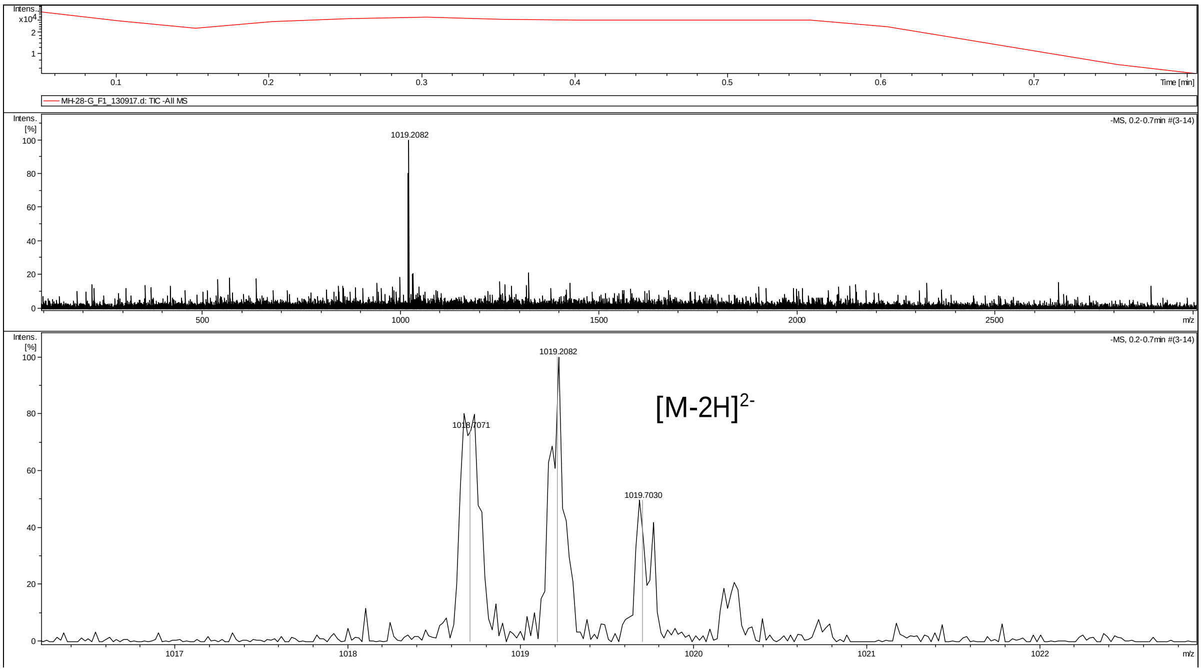

Figure S8. Mass spectrum of oligonucleotide ON3b.

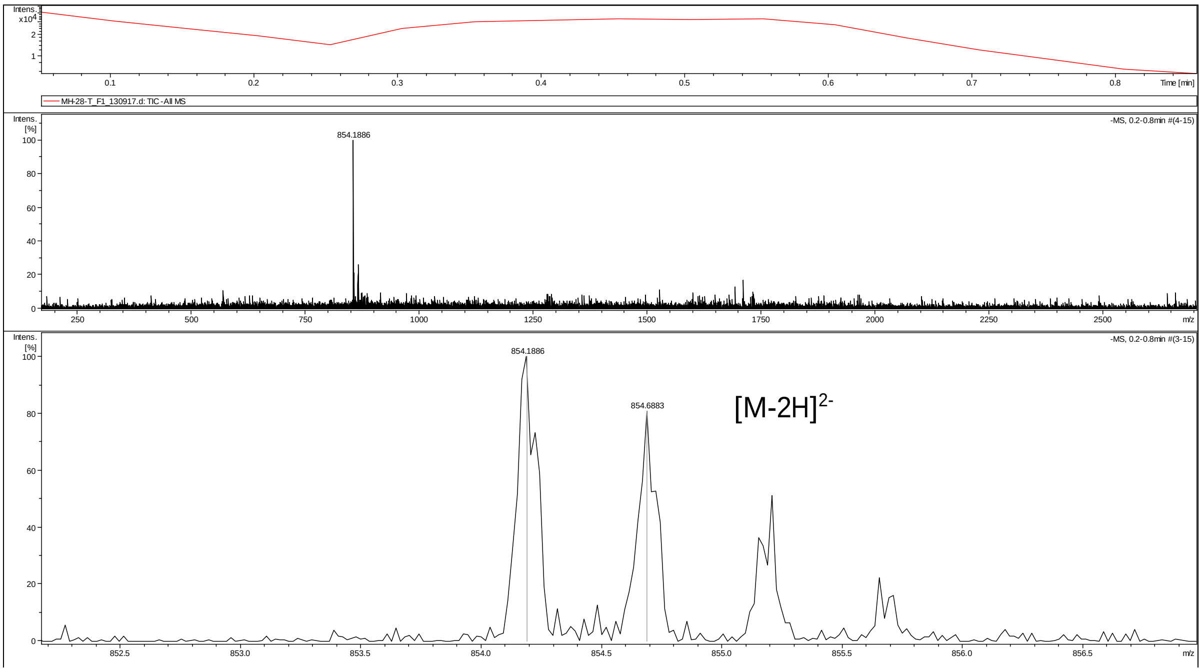

Figure S9. Mass spectrum of oligonucleotide ON4b.

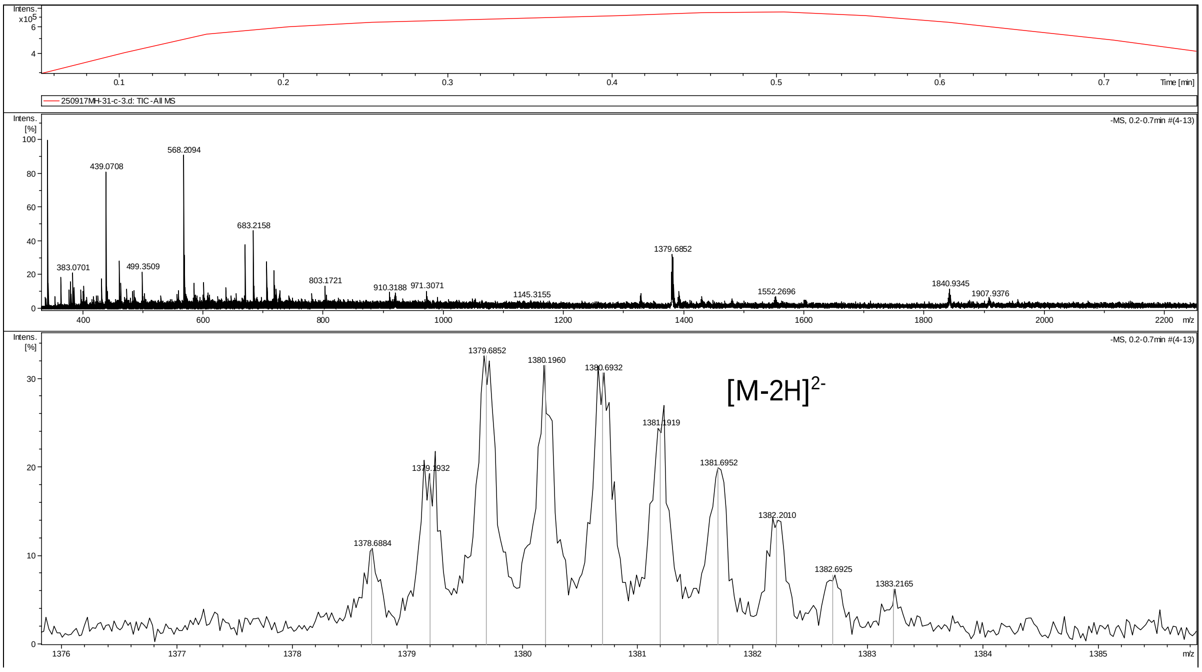

Figure S10. Mass spectrum of oligonucleotide ON1b-Pd.

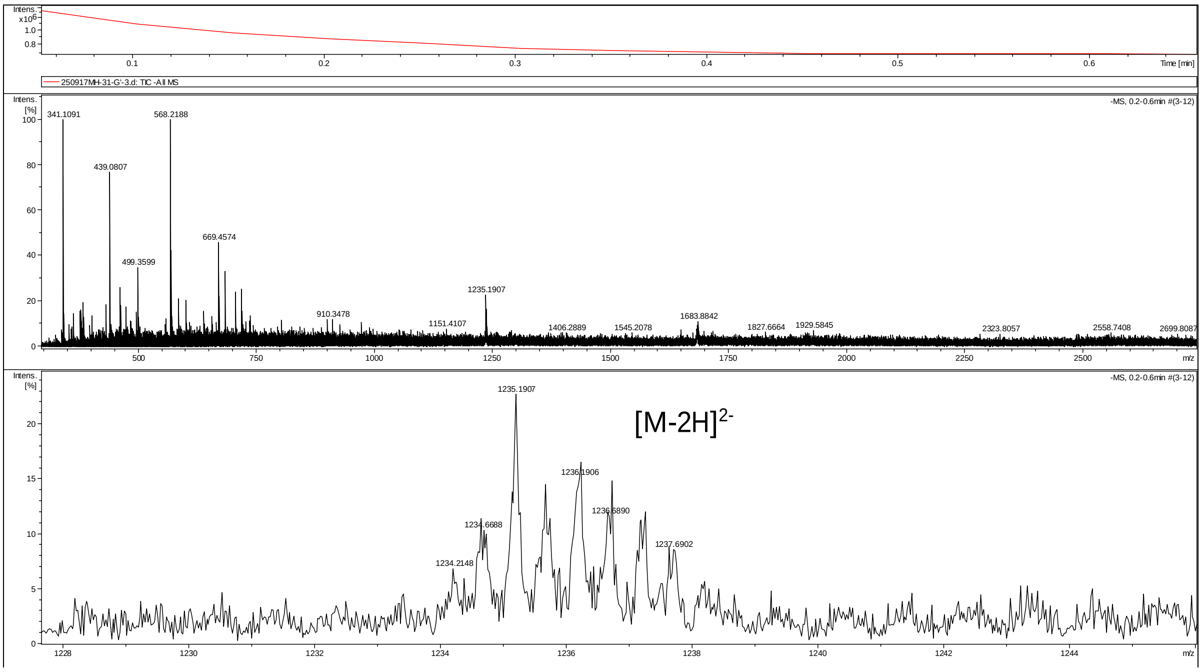

Figure S11. Mass spectrum of oligonucleotide ON2b-Pd.

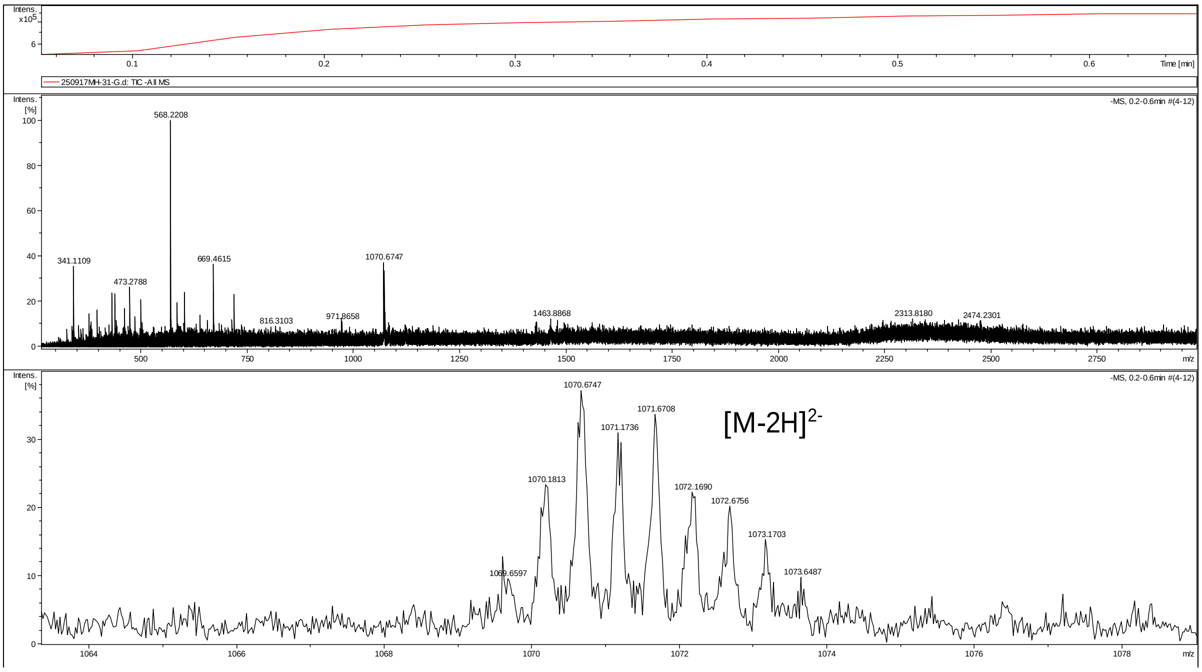

Figure S12. Mass spectrum of oligonucleotide ON3b-Pd.

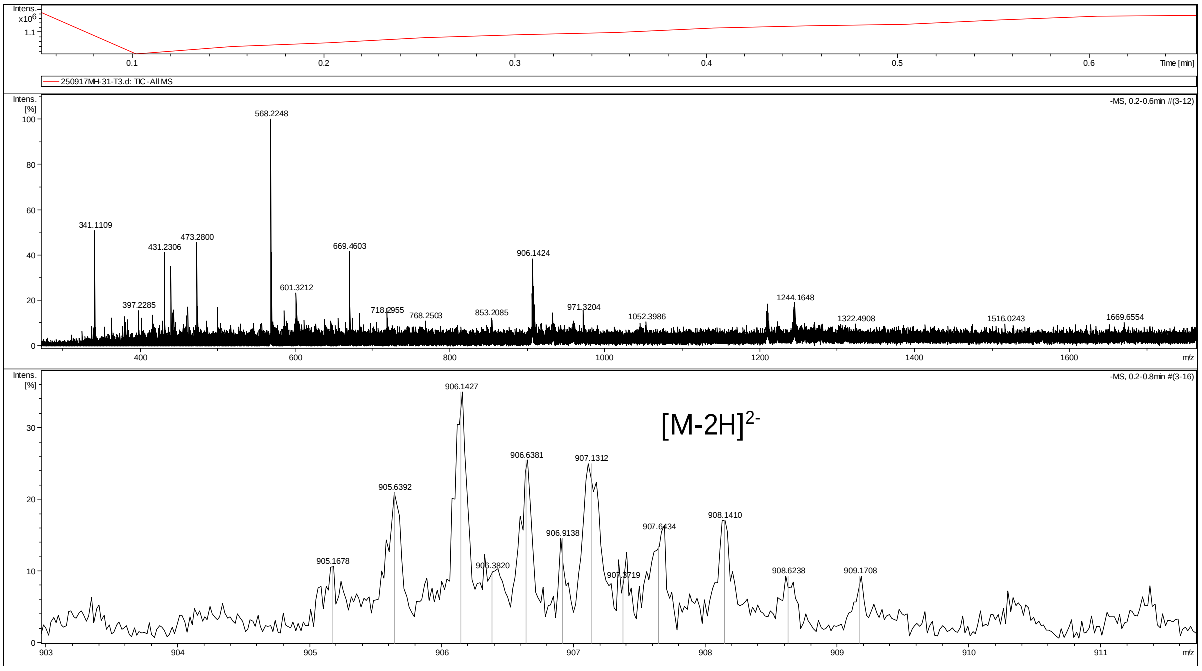

Figure S13. Mass spectrum of oligonucleotide ON4b-Pd.

Table S1. Melting temperatures of duplexes formed by ON1a, ON1b, ON1b-Pd, ON2a, ON2b, ON2b-Pd, ON3a, ON3b, ON3b-Pd, ON4a, ON4b and ON4b-Pd with ON5a, ON5c, ON5g and ON5t; pH = 7.4 (20 mM cacodylate buffer); [oligonucleotides] = 3.0 μM; I($NaClO_4$) = 0.10 M.

	T_m / °C			
	ON5a	ON5c	ON5g	ON5t
ON1a	40.4 ± 0.7	n.a. [1]	17.6 ± 0.4	17.9 ± 0.3
ON1b	35.8 ± 0.6	n.a. [1]	16.0 ± 0.7	16.9 ± 0.4
ON1b-Pd	41.0 ± 0.1	n.a. [1]	22.6 ± 0.4	22.4 ± 0.7
ON2a	31.8 ± 1.3	n.a. [1]	n.a. [1]	n.a. [1]
ON2b	30.5 ± 1.3	n.a. [1]	n.a. [1]	n.a. [1]
ON2b-Pd	32.7 ± 1.0	n.a. [1]	n.a. [1]	n.a. [1]
ON3a	21.0 ± 0.9	n.a. [1]	n.a. [1]	n.a. [1]
ON3b	20.5 ± 0.8	n.a. [1]	n.a. [1]	n.a. [1]
ON3b-Pd	21.2 ± 1.2	n.a. [1]	n.a. [1]	n.a. [1]
ON4a	16.1 ± 0.4	n.a. [1]	n.a. [1]	n.a. [1]
ON4b	15.4 ± 0.8	n.a. [1]	n.a. [1]	n.a. [1]
ON4b-Pd	15.7 ± 0.8	n.a. [1]	n.a. [1]	n.a. [1]

[1] No sigmoidal melting curve was obtained.

Table S2. Melting temperatures of duplexes formed by ON1a, ON1b, ON1b-Pd, ON2a, ON2b, ON2b-Pd, ON3a, ON3b, ON3b-Pd, ON4a, ON4b and ON4b-Pd with ON5a, ON5c, ON5g and ON5t in the presence of 2-mercaptoethanol; pH = 7.4 (20 mM cacodylate buffer); [oligonucleotides] = 3.0 μM; [2-mercaptoethanol] = 100 μM; I($NaClO_4$) = 0.10 M.

	T_m / °C			
	ON5a	ON5c	ON5g	ON5t
ON1a	40.8 ± 0.6	n.a. [1]	16.2 ± 0.7	15.5 ± 0.3
ON1b	34.9 ± 1.1	n.a. [1]	16.8 ± 0.6	15.5 ± 1.1
ON1b-Pd	38.3 ± 0.9	n.a. [1]	17.3 ± 0.8	15.3 ± 0.7
ON2a	30.9 ± 1.2	n.a. [1]	n.a. [1]	n.a. [1]
ON2b	29.3 ± 0.8	n.a. [1]	n.a. [1]	n.a. [1]
ON2b-Pd	30.2 ± 0.1	n.a. [1]	n.a. [1]	n.a. [1]
ON3a	19.0 ± 0.5	n.a. [1]	n.a. [1]	n.a. [1]
ON3b	20.0 ± 0.9	n.a. [1]	n.a. [1]	n.a. [1]
ON3b-Pd	19.3 ± 0.3	n.a. [1]	n.a. [1]	n.a. [1]
ON4a	17.3 ± 1.0	n.a. [1]	n.a. [1]	n.a. [1]
ON4b	17.0 ± 0.8	n.a. [1]	n.a. [1]	n.a. [1]
ON4b-Pd	17.6 ± 0.9	n.a. [1]	n.a. [1]	n.a. [1]

[1] No sigmoidal melting curve was obtained.

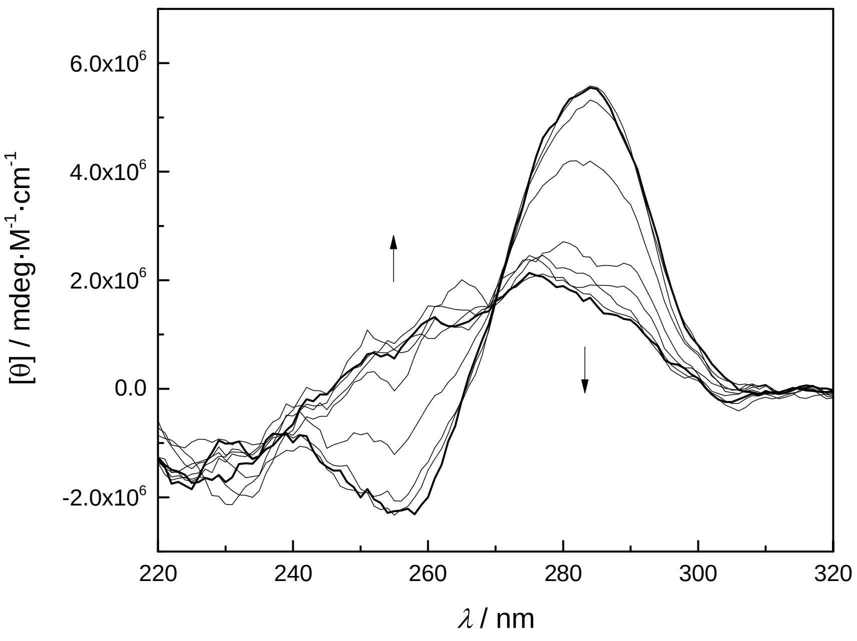

Figure S14. CD spectra of ON1b-Pd•ON5a, recorded at 10 °C intervals between 10 and 90 °C; pH = 7.4 (20 mM cacodylate buffer); [oligonucleotides] = 3.0 μM; I($NaClO_4$) = 0.10 M. Spectra acquired at extreme temperatures are indicated by thicker lines and thermal shifts of the minima and maxima by arrows.

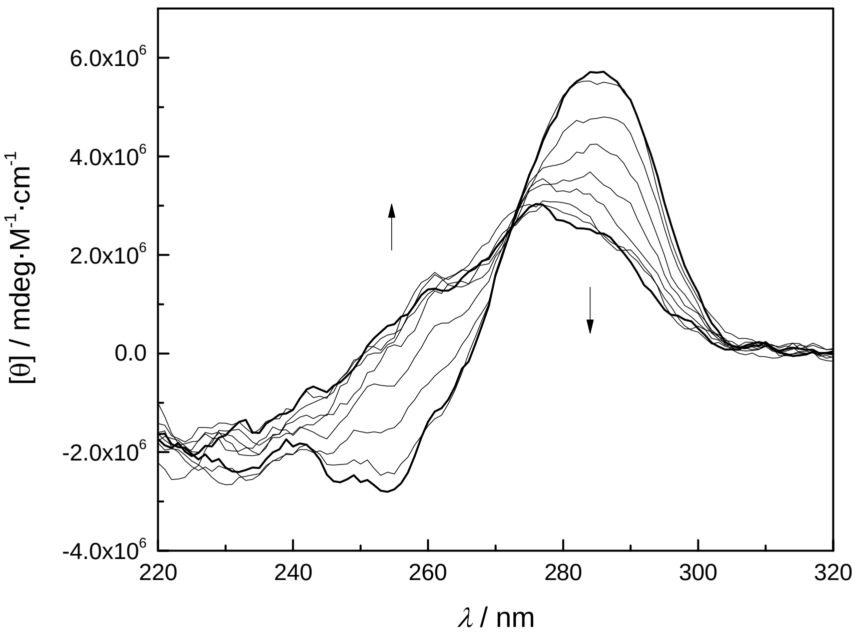

Figure S15. CD spectra of ON1b-Pd•ON5c, recorded at 10 °C intervals between 10 and 90 °C; pH = 7.4 (20 mM cacodylate buffer); [oligonucleotides] = 3.0 μM; I($NaClO_4$) = 0.10 M. Spectra acquired at extreme temperatures are indicated by thicker lines and thermal shifts of the minima and maxima by arrows.

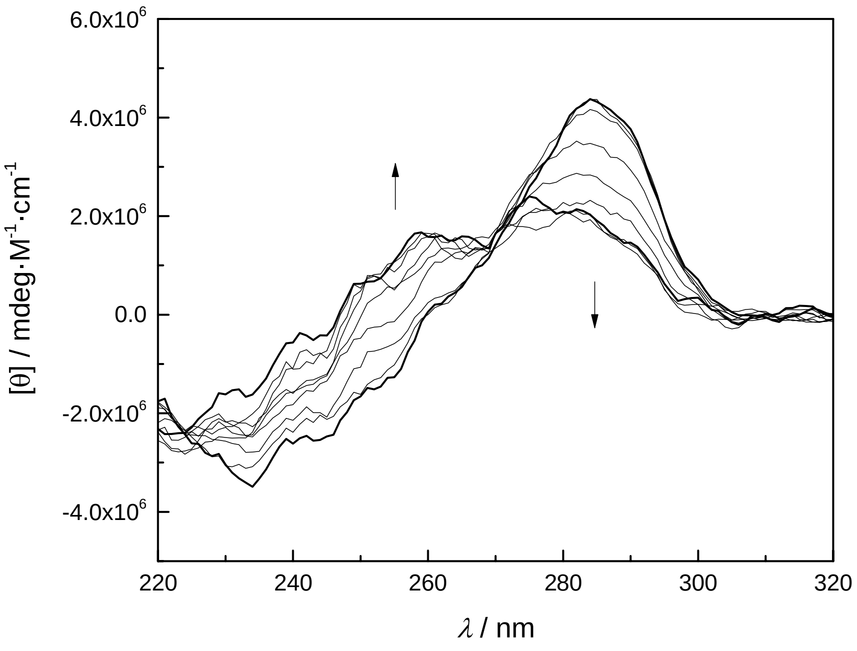

Figure S16. CD spectra of ON1b-Pd•ON5g, recorded at 10 °C intervals between 10 and 90 °C; pH = 7.4 (20 mM cacodylate buffer); [oligonucleotides] = 3.0 μM; I($NaClO_4$) = 0.10 M. Spectra acquired at extreme temperatures are indicated by thicker lines and thermal shifts of the minima and maxima by arrows.

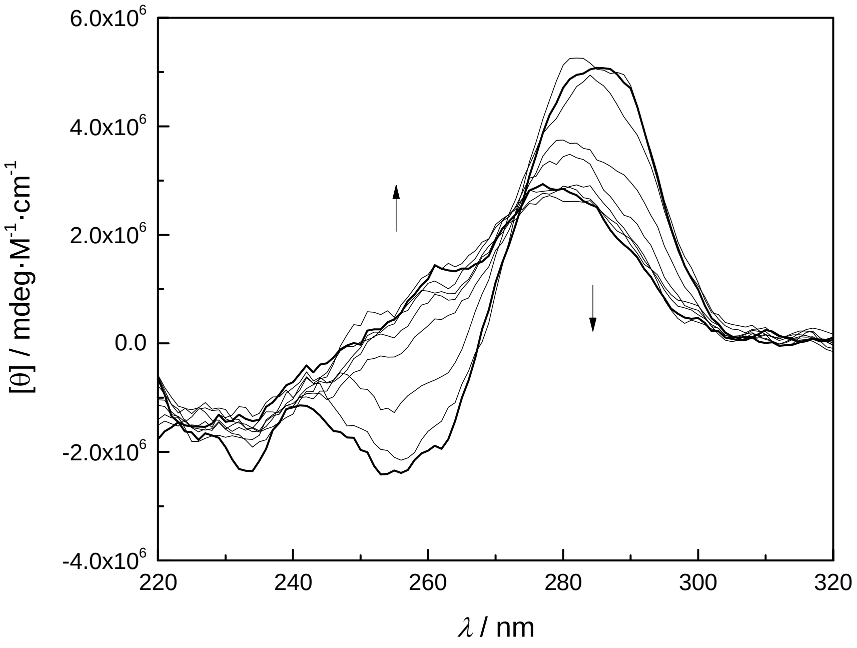

Figure S17. CD spectra of ON1b-Pd•ON5t, recorded at 10 °C intervals between 10 and 90 °C; pH = 7.4 (20 mM cacodylate buffer); [oligonucleotides] = 3.0 μM; I($NaClO_4$) = 0.10 M. Spectra acquired at extreme temperatures are indicated by thicker lines and thermal shifts of the minima and maxima by arrows.

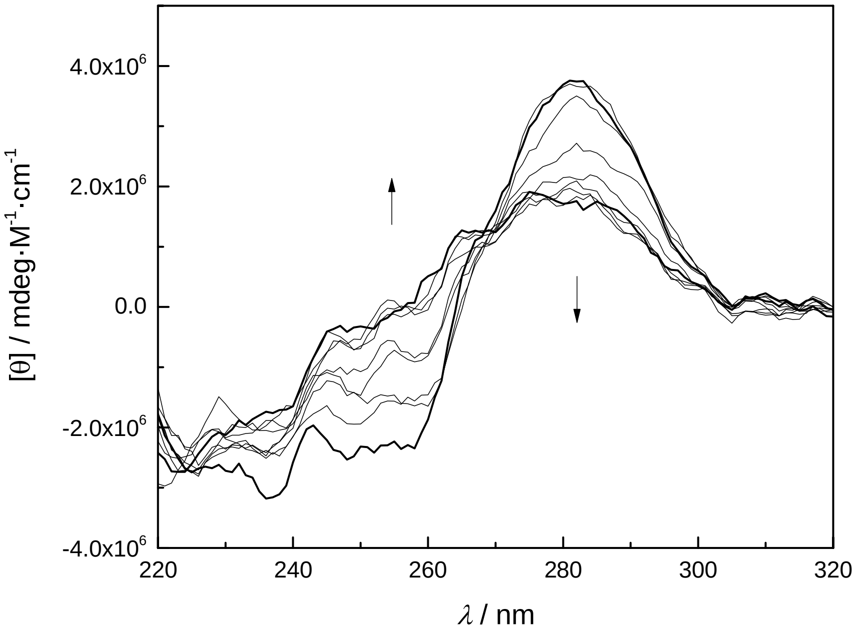

Figure S18. CD spectra of ON2b-Pd•ON5a, recorded at 10 °C intervals between 10 and 90 °C; pH = 7.4 (20 mM cacodylate buffer); [oligonucleotides] = 3.0 μM; I($NaClO_4$) = 0.10 M. Spectra acquired at extreme temperatures are indicated by thicker lines and thermal shifts of the minima and maxima by arrows.

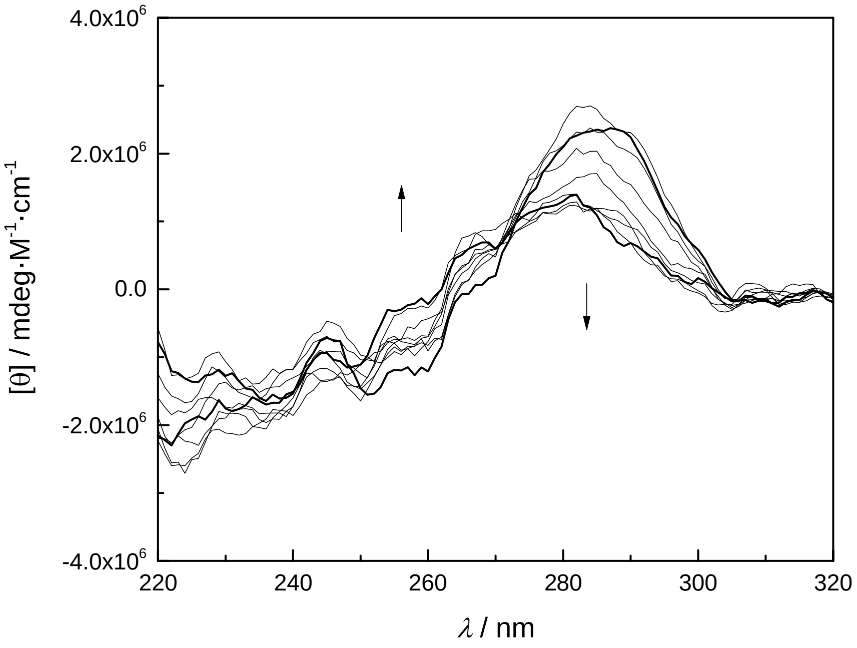

Figure S19. CD spectra of ON2b-Pd•ON5c, recorded at 10 °C intervals between 10 and 90 °C; pH = 7.4 (20 mM cacodylate buffer); [oligonucleotides] = 3.0 μM; I($NaClO_4$) = 0.10 M. Spectra acquired at extreme temperatures are indicated by thicker lines and thermal shifts of the minima and maxima by arrows.

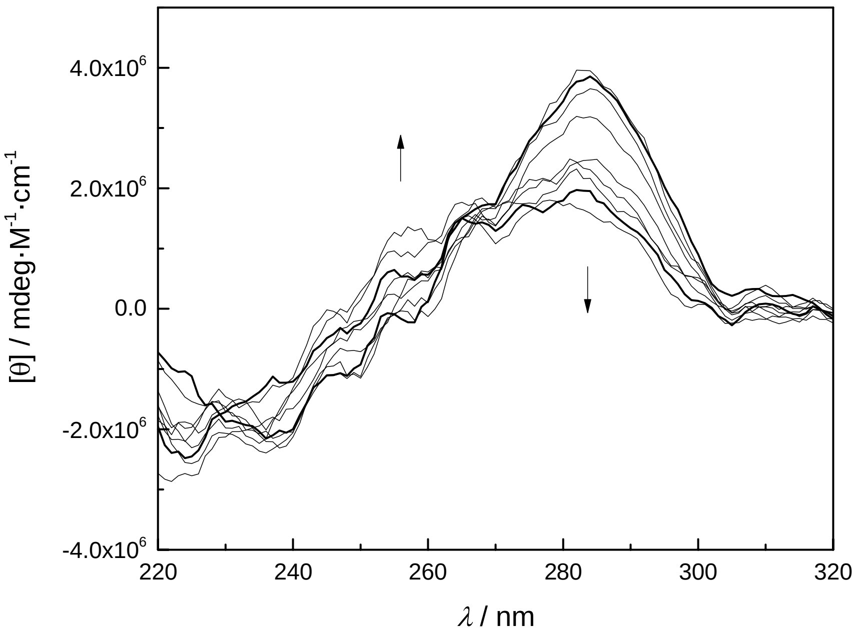

Figure S20. CD spectra of ON2b-Pd•ON5g, recorded at 10 °C intervals between 10 and 90 °C; pH = 7.4 (20 mM cacodylate buffer); [oligonucleotides] = 3.0 μM; I($NaClO_4$) = 0.10 M. Spectra acquired at extreme temperatures are indicated by thicker lines and thermal shifts of the minima and maxima by arrows.

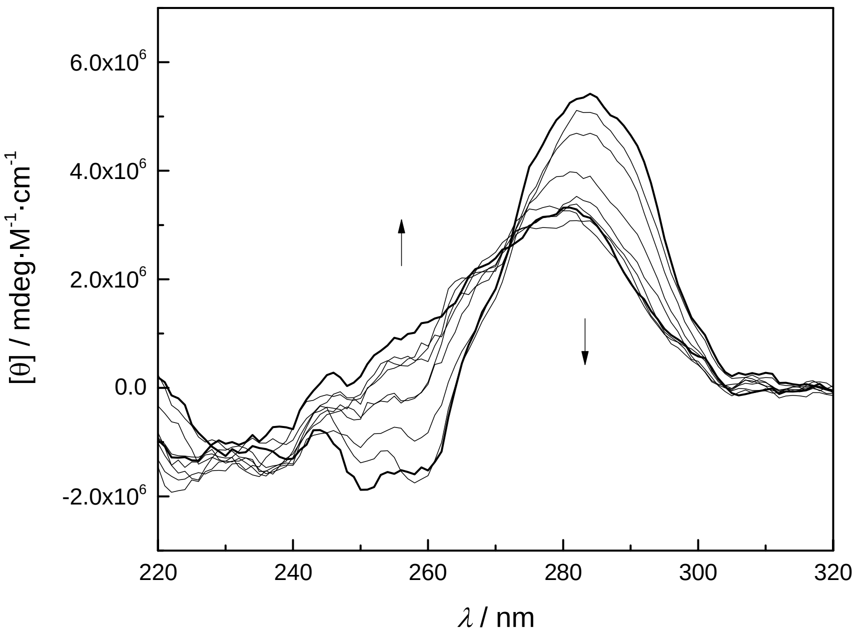

Figure S21. CD spectra of ON2b-Pd•ON5t, recorded at 10 °C intervals between 10 and 90 °C; pH = 7.4 (20 mM cacodylate buffer); [oligonucleotides] = 3.0 μM; I($NaClO_4$) = 0.10 M. Spectra acquired at extreme temperatures are indicated by thicker lines and thermal shifts of the minima and maxima by arrows.

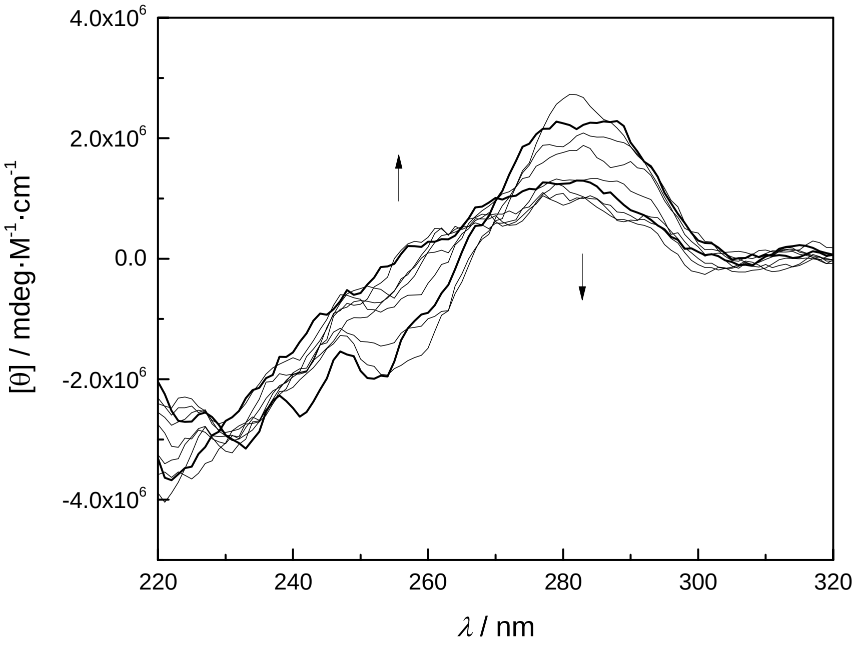

Figure S22. CD spectra of ON3b-Pd•ON5a, recorded at 10 °C intervals between 10 and 90 °C; pH = 7.4 (20 mM cacodylate buffer); [oligonucleotides] = 3.0 μM; I($NaClO_4$) = 0.10 M. Spectra acquired at extreme temperatures are indicated by thicker lines and thermal shifts of the minima and maxima by arrows.

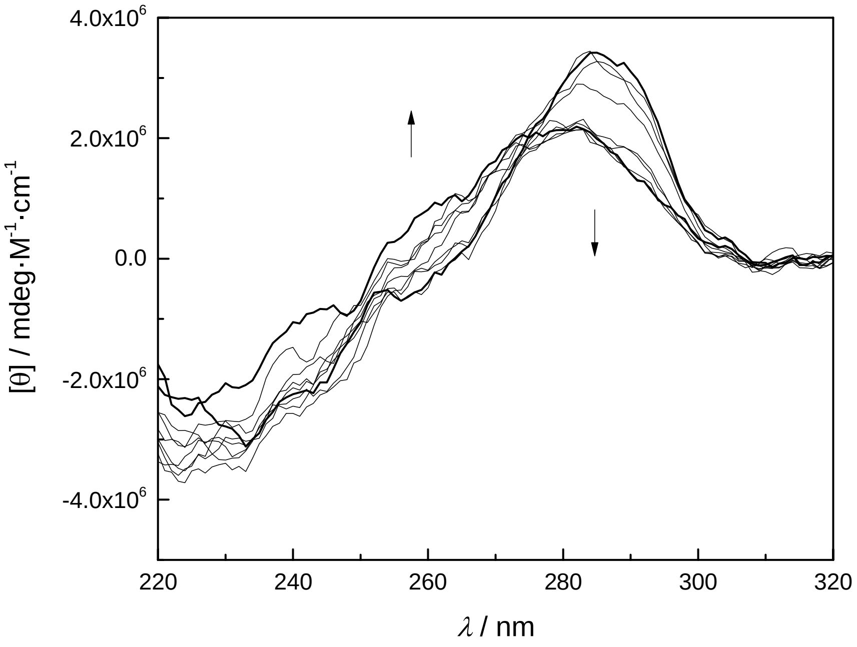

Figure S23. CD spectra of ON3b-Pd•ON5c, recorded at 10 °C intervals between 10 and 90 °C; pH = 7.4 (20 mM cacodylate buffer); [oligonucleotides] = 3.0 μM; I($NaClO_4$) = 0.10 M. Spectra acquired at extreme temperatures are indicated by thicker lines and thermal shifts of the minima and maxima by arrows.

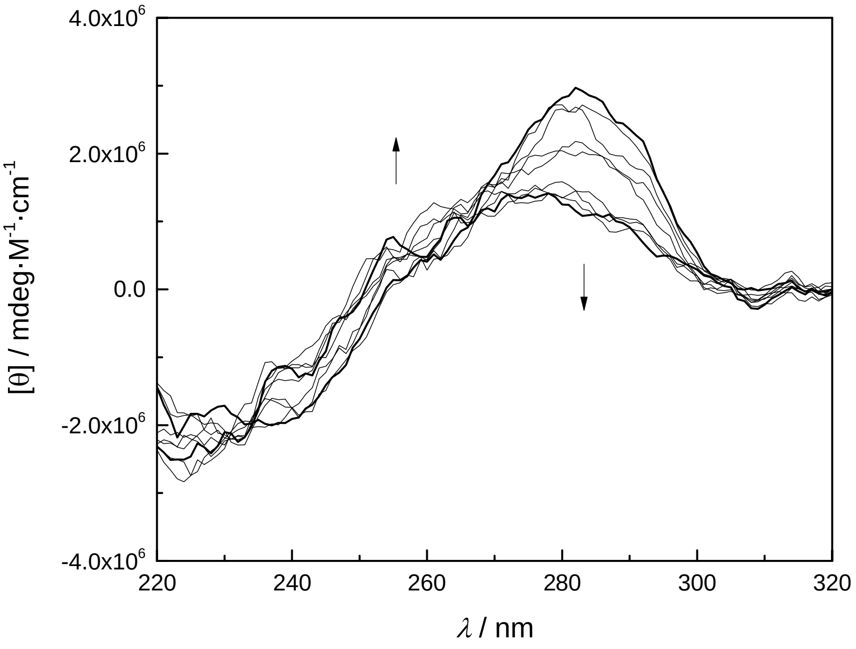

Figure S24. CD spectra of ON3b-Pd•ON5g, recorded at 10 °C intervals between 10 and 90 °C; pH = 7.4 (20 mM cacodylate buffer); [oligonucleotides] = 3.0 μM; I($NaClO_4$) = 0.10 M. Spectra acquired at extreme temperatures are indicated by thicker lines and thermal shifts of the minima and maxima by arrows.

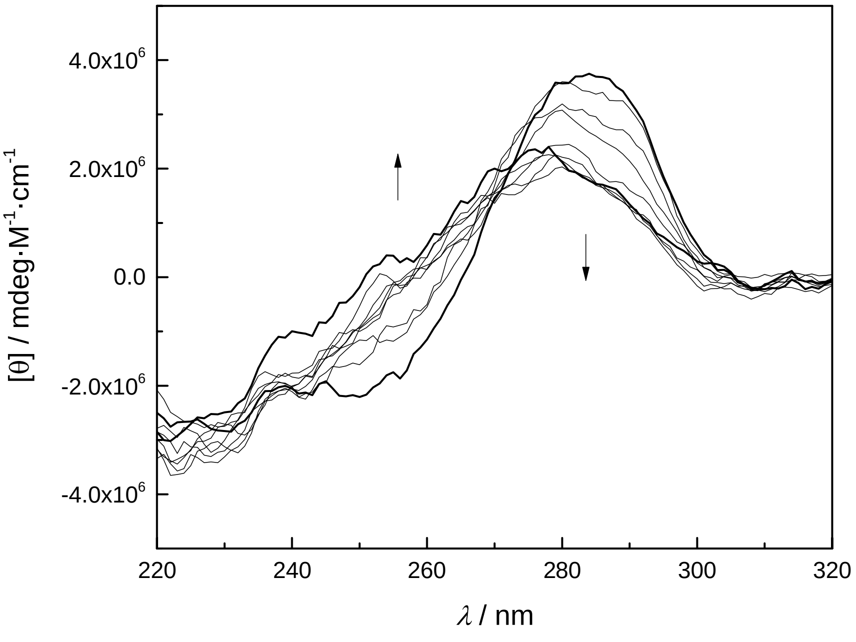

Figure S25. CD spectra of ON3b-Pd•ON5t, recorded at 10 °C intervals between 10 and 90 °C; pH = 7.4 (20 mM cacodylate buffer); [oligonucleotides] = 3.0 μM; I($NaClO_4$) = 0.10 M. Spectra acquired at extreme temperatures are indicated by thicker lines and thermal shifts of the minima and maxima by arrows.

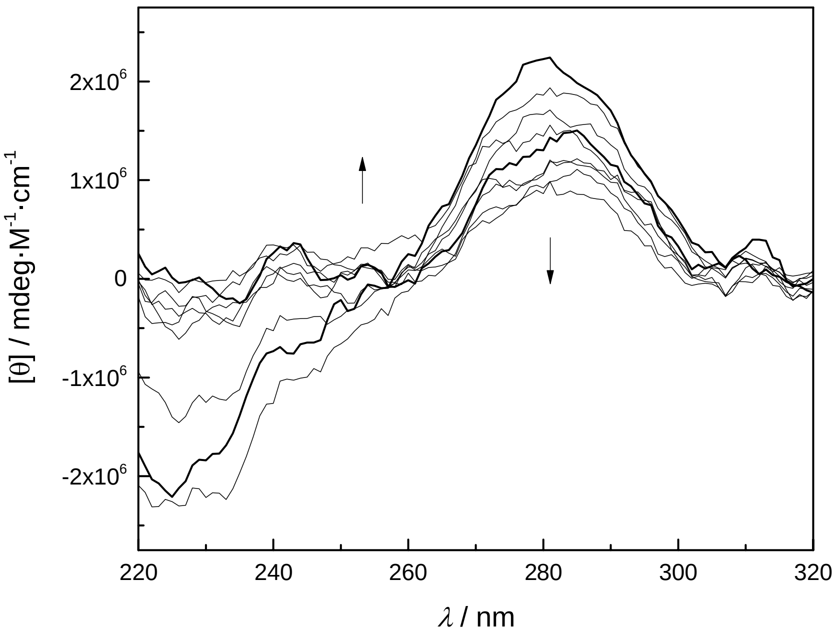

Figure S26. CD spectra of ON4b-Pd•ON5a, recorded at 10 °C intervals between 10 and 90 °C; pH = 7.4 (20 mM cacodylate buffer); [oligonucleotides] = 3.0 μM; I($NaClO_4$) = 0.10 M. Spectra acquired at extreme temperatures are indicated by thicker lines and thermal shifts of the minima and maxima by arrows.

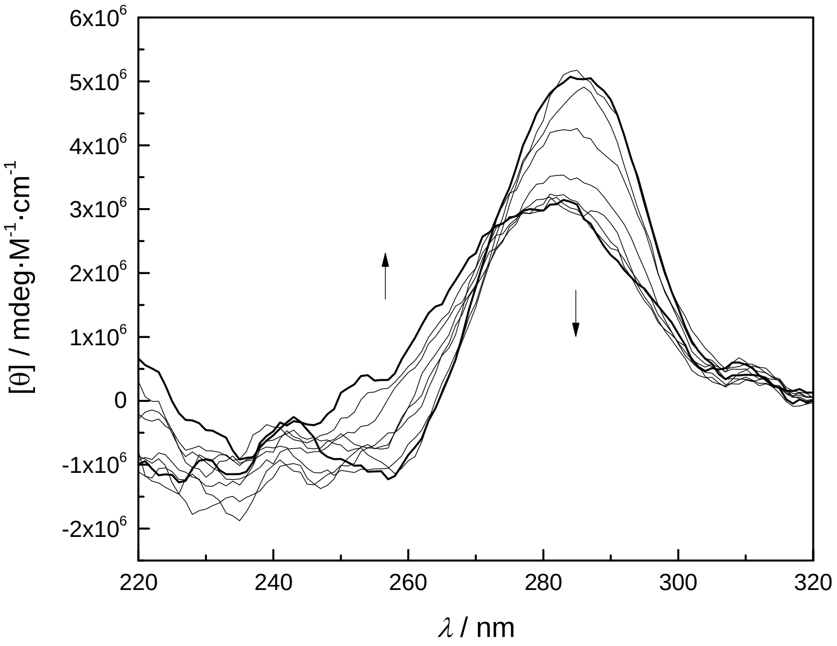

Figure S27. CD spectra of ON4b-Pd•ON5c, recorded at 10 °C intervals between 10 and 90 °C; pH = 7.4 (20 mM cacodylate buffer); [oligonucleotides] = 3.0 μM; I($NaClO_4$) = 0.10 M. Spectra acquired at extreme temperatures are indicated by thicker lines and thermal shifts of the minima and maxima by arrows.

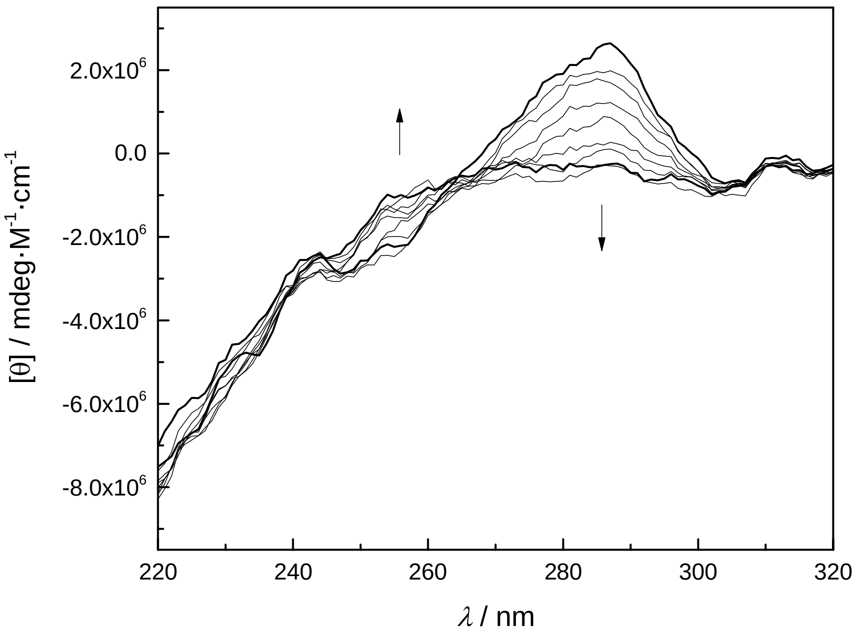

Figure S28. CD spectra of ON4b-Pd•ON5g, recorded at 10 °C intervals between 10 and 90 °C; pH = 7.4 (20 mM cacodylate buffer); [oligonucleotides] = 3.0 μM; I($NaClO_4$) = 0.10 M. Spectra acquired at extreme temperatures are indicated by thicker lines and thermal shifts of the minima and maxima by arrows.

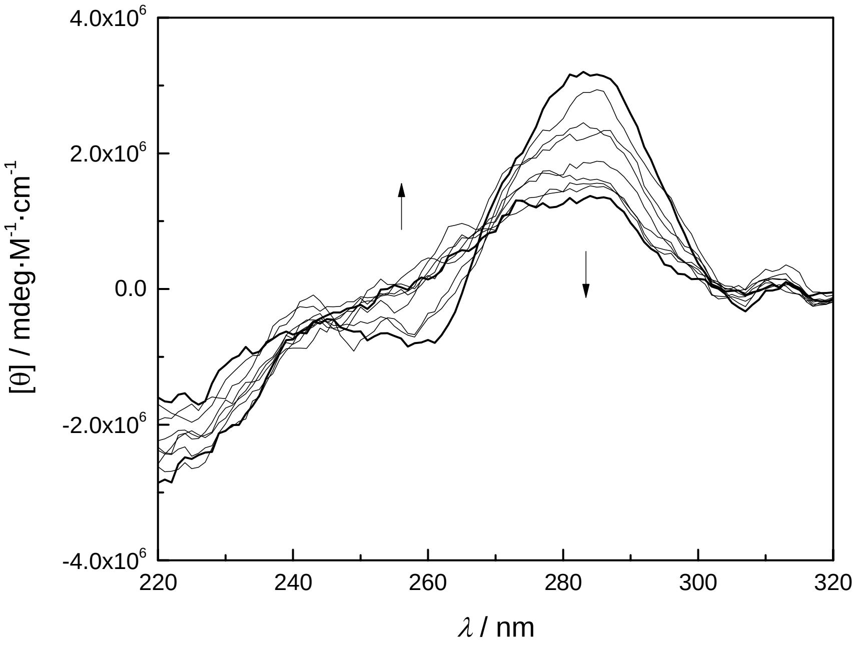

Figure S29. CD spectra of ON4b-Pd•ON5t, recorded at 10 °C intervals between 10 and 90 °C; pH = 7.4 (20 mM cacodylate buffer); [oligonucleotides] = 3.0 μM; I($NaClO_4$) = 0.10 M. Spectra acquired at extreme temperatures are indicated by thicker lines and thermal shifts of the minima and maxima by arrows.
